# Hypoglycemic and hypolipidemic effects of total saponins from *Stauntonia chinensis* in diabetic *db/db* mice

**DOI:** 10.1111/jcmm.13876

**Published:** 2018-10-16

**Authors:** Jing Xu, Sha Wang, Tianhui Feng, Yu Chen, Guangzhong Yang

**Affiliations:** ^1^ School of Pharmaceutical Sciences South‐Central University for Nationalities Wuhan China; ^2^ College of Chemistry and Material Sciences South‐Central University for Nationalities Wuhan China; ^3^ National Demonstration Center for Experimental Ethnopharmacology Education South‐Central University for Nationalities Wuhan China

**Keywords:** AMPK/ACC signalling pathway, hypoglycemic effect, hypolipidemic effect, IRS‐1/PI3K/Akt signalling pathway, *Stauntonia chinensis*, triterpene saponins

## Abstract

*Stauntonia chinensis* DC. has been utilised as a traditional herbal medicine for its anti‐hyperglycemic characteristic, which has been associated with triterpene saponins. The goal of the current evaluation was to examine hypoglycemic activity and affiliated mechanism of total saponins from *S. chinensis*. The chemical composition was analysed by HPLC‐ESI‐MS/MS. The fasting blood glucose, oral glucose tolerance test, insulin tolerance test, insulin and glycogen levels of type 2 diabetic *db/db* mice administered total saponins were quantified to determine the hypoglycemic effects. The serum lipid profiles were assessed to determine the hypolipidemic effects. Western blotting was used to quantify the protein levels of insulin receptor substrates (IRS)‐1/PI3K/AKT, AMPK/ACC and GLUT4. Twenty triterpene saponins were identified from the total saponins, which exhibited hypoglycemic activities and modulated hyperlipidemia that was associated with type 2 diabetes. The hypoglycemic effects were partly due to the activation of GLUT4, which is regulated by IRS‐1/PI3K/AKT. The activation of the AMPK/ACC signalling pathway may be responsible for the hypolipidemic activity. This study revealed that total saponins from *S. chinensis* have significant hypoglycemic and hypolipidemic activity in diabetic *db/db* mice, indicating that these may be utilised in the development of saponins based on *S. chinensis* for the treatment of type 2 diabetes.

## 1 INTRODUCTION

Type 2 diabetes mellitus (T2DM) is a persistent metabolic disorder that is distinguished by postprandial and fasting hyperglycemia due to insulin resistance.^1^ According to the World Health Organization (WHO), nearly 415 million people had diabetes in 2016, with T2DM responsible for >90% of these cases.^2^, ^3^ Further, the majority of T2DM cases are connected with high blood lipid profiles, which can result in metabolic syndrome and later cause other medical issues, including cardiovascular diseases.^4^

The contemporary strategy to treat T2DM primarily involves oral anti‐hyperglycemic drugs, including sulfonylureas, biguanides α‐glucosidase inhibitors and thiazolidinedione. Nevertheless, these synthetic therapeutics cannot entirely regulate the glycemic levels and have unfavorable side effects, such as flatulence, nausea, meteorism and potentially diarrhea.^5^, ^6^ Natural compounds obtained from plant and agricultural crop resources have been utilised for diabetes because of their limited side effects.^7^

Saponins are plant secondary metabolites with wide pharmacological and industrial applications. Plant species containing saponins (*Panax ginseng*,* Quillaja saponaria* and *Aesculus hippocas‐tanum*) as well as isolated compounds (asiaticoside and aescin) have been widely used in the pharmaceutical and cosmetic industries.^8^ Pharmacological studies have revealed that saponins exhibit properties that reduce elevated plasma blood glucose, regulate lipid metabolism and reduce body fat without the occurrence of significant adverse health effects.^9^ It has been reported that saponins exert an anti‐hyperglycemic function by increasing plasma insulin levels,^10^ restoring insulin response,^11^ activating glycogen synthesis,^12^ and inhibiting α‐glucosidase activity.^13^ Saponins have also lowered increased triglyceride and serum cholesterolmodulates linked to T2DM by regulating the expression of numerous genes connected to lipid metabolism.^9^ These capabilities make saponins an outstanding drug candidate for the treatment of T2DM.

*Stauntonia chinensis* DC., frequently referred to as “Ye Mu Gua,” is an evergreen herb that grows throughout Southern China and is a part of the Lardizabalaceae family. *S. chinensis* has been utilised as a traditional herbal medicine due to its anti‐inflammatory, anti‐hyperglycemic and analgesic characteristics.^14^ The previous phytochemical study on *S. chinensis* indicated that it is a rich source of saponins, which may be responsible for the pharmacological activities of this plant.^15^, ^16^ The results of our previous study showed that triterpene saponins from *S. chinensis* improved glucose uptake and insulin sensitivity in hepatic cells.^17^ However, the hypoglycemic effect *in vivo* and the underlying mechanism remained unclear. For the development of the saponins based on *S. chinensis* for medicinal use, we investigated the triterpene saponin structures, hypoglycemic and hypolidemic effects and underlying mechanism of the total saponins from *S. chinensis* (TSS) in type 2 diabetic *db/db* mice.

## 2 MATERIALS AND METHODS

### 2.1 Chemicals and reagents

Each of the chemical reagents and the HP‐20 micro resin were of analytical grade and purchased from Sinopharm (Shanghai, China). Glycine, bovine insulin, sodium dodecyl sulfate, sodium salt (SDS), glycerine, tween‐20, ammonium persulfate and skimmed milk powder and dimethyl sulfoxide were purchased from BioSharp (Hefei, Anhui, China). Trypsogen, acrylamide, Tris‐base and tetramethyl ethylene diamine were purchased from Sigma (St. Louis, MO, USA). Glucose and a glycogen assay kit were purchased from Jiancheng (Nanjing, Jiangsu, China). Protein lysis buffer and the molecular weight marker were obtained from Thermo Fisher Scientific (Waltham, MA, USA). All of the antibodies were purchased from Santa Cruz Biotechnology (Santa Cruz, CA, USA).

### 2.2 Plant materials

The stems of *S. chinensis* were gathered from Nanning, Guangxi Zhuang Autonomous Region, P. R. China and discerned by Jin‐Wei Huang, an associate chief pharmacist of Guangxi Institute of Minority Medicine. The voucher specimen (20090801) was submitted to the Herbarium of College of Pharmacy, South‐Central University for Nationalities.

### 2.3 Preparation of TSS

The stems of *S. chinensis* (2.5 kg) were removed with 60% ethanol (EtOH) three times and then consecutively separated with EtOAc and *n*‐BuOH. The extract of *n*‐BuOH (109 g) was subjected to HP‐20 micro resin and eluted with water, 30% aqueous ethanol, 50% aqueous ethanol and 70% aqueous ethanol to produce five fractions. The TSS was acquired from the 50% and 70% ethanol fractions and saponin content of up to 90.48% was quantified using the colorimetric method.^18^

### 2.4 HPLC conditions

Chromatography was conducted on a Thermo Scientific Dionex UltiMate 3000 UHPLC^+^ Focused LC system (Thermo Fisher Scientific). The columns were kept at 30°C. The separation was performed on a Thermo Scientific Hypersil ODS C18 column (2.1 mm × 150 mm, i.d., 3 μm; Thermo Fisher Scientific). The mobile phase used water with 0.1% (v/v) formic acid (A) and acetonitrile with 0.1% (v/v) formic acid (B). A gradient program was utilised as follows: 0‐40 minutes, 20%‐40% B; 40‐60 minutes, 40%‐60% B; 60‐70 minutes, 60%‐85% B; the composition then remained at 85% B for 3 minutes and returned to the initial conditions and was kept at 10 minutes for equilibration. The flow rate was 0.2 mL/min.

### 2.5 Mass spectrometry conditions

MS was conducted with an LCQ Fleet ion trap mass spectrometer (Thermo Fisher Scientific) through an ESI interface, which was operated in the positive and negative ion modes. The conditions of the ESI source were as follows: ispray voltage 5 kV; spray current 10 μA; sheath gas flow rate 35 arbitrary units; Aux gas flow rate 10 arbitrary units; sweep gas flow rate 1 arbitrary units; capillary temperature 350°C; capillary voltage 10 V; tube lens 90 V; and the mass range was recorded *m/z* 100‐2000. The chemicals were draw by software “Chemdraw.”

### 2.6 Animals

Six‐week‐old C57 *db/db* male mice were obtained from the Model Animal Research Center of Nanjing University (Nanjing, Jiangsu, China). Each mouse was kept at a regulated room temperature (25 ± 2°C), humidity (55 ± 5%) and a 12 hour/12 hour light/dark cycle and the mice were provided with water and typical laboratory feed ad libitum. Each mouse was taken care of in accordance with the Animal Experimental Ethics Committee of South‐Central University for Nationalities (Approval no. 2016‐SCUEC‐AEC‐0037). All efforts were made to limit the animals’ suffering.

### 2.7 Acute toxicity study

Kunming mice (n = 50) of either sex weighing 18‐22 g were subjected to an acute toxicity study. The doses of TSS for the study were 0.5, 1, 1.5 and 2 g/kg bodyweight (BW). After food and water deprivation for 12 hours, the mice were treated orally with 0.9% saline or TSS dissolved in 0.9% saline. The mice were examined at 0 minute, 15 minutes, 30 minutes, 60 minutes, 120 minutes, 180 minutes and 24 hours following the TSS treatment for behavioural, neurological and autonomic profiles and lethality.

### 2.8 Treatment protocol

After 2 weeks of adaptive breeding, the mice were randomly assigned to six groups. C57 male mice (n = 6) were set as the standard chow control group (Normal), and C57 *db/db* male mice were divided into five groups containing six mice each: the T2DM model *db/db* group (Model), the 200 mg/kg metformin positive control group (Met), the 30 mg/kg TSS group (TSS‐30 mg/kg), the 60 mg/kg TSS group (TSS‐60 mg/kg), and the 120 mg/kg TSS group (TSS‐120 mg/kg). All of the mice were dosed by gavage once a day for three consecutive weeks and weighed every 3 days throughout the experiment.

### 2.9 Preparation and biochemical analysis of serum samples

The mice were fasted for 12 hours and blood samples were obtained from the tail vein. The fasting blood glucose (FBG) was quantified with a OneTouch Ultra glucose monitor (Johnson, TX, USA) prior to the beginning of the treatment (day 0) and every 7 days throughout the experiment. The serum was divided by centrifugation and subjected to the quantification of various biochemical parameters: total cholesterol (TC), triglyceride (TG), high/low‐density lipoprotein‐cholesterol (H/LDL‐C), and insulin. The Cholesterol, LDL‐C and HDL‐C were evaluated by using an automatic biochemical analyser (Hitachi 7180+ISE, Tokyo, Japan). Serum insulin levels were determined by Elisa kits (Mercodia, Uppsala, Sweden).

On day 15 of the treatment, an oral glucose tolerance test (OGTT) was conducted following an overnight fast. The blood glucose concentration before glucose administration was set as baseline (0 minute). After that, the mice were orally administered 2 g glucose/kg of BW, and the blood glucose level was established at 30, 60 and 120 minutes following oral glucose administration.

An insulin tolerance test (ITT) was conducted on day 18 of the treatment after overnight fasting. The blood glucose levels were quantified prior to injecting insulin (0 minute) and at 30, 60 and 120 minutes following the inter‐peritoneal injection of insulin (0.2 U/kg BW).

### 2.10 Determination of liver and skeletal glycogen levels

At the end of the treatment, the mice were killed with an overdose of intraperitoneal anaesthesia. Tissue samples from the liver and skeletal muscles were removed, rinsed with 0.9% saline, weighed and kept at −80°C for additional analysis. The glycogen contents of the liver and skeletal muscle were quantified with a typical glycogen kit (Jiancheng, Nanjing, Jiangsu Province, China) following the manufacturer's recommendations. The amount of glycogen in every tissue sample was expressed as milligrams of glucose per gram of tissue.

### 2.11 Histopathological examination

The liver tissues were excised and washed with normal saline. The washed tissues were immediately fixed in 10% neutral‐buffered formalin solution and processed via the paraffin wax embedding method. The paraffin‐embedded tissues were cut into 5 μm thickness section followed by hematoxylin‐eosin staining (HE) for histopathological examination.

### 2.12 Western blot analysis

Total proteins from the liver and skeletal muscle were extracted and quantified using a BCA protein assay kit (Beyotime, Shanghai, China). The proteins were divided with 10% sodium dodecyl sulfate‐polyacrylamide gel electrophoresis (SDS‐PAGE) and moved to polyvinylidene difluoride membranes (Millipore, Shanghai, China). The membranes were halted by 5% skimmed milk/BSA for 2 hours at room temperature and incubated with monoclonal antibodies overnight at 4°C. After that, the membranes were rinsed and incubated with secondary antibodies for 2 hours at room temperature. The protein bands were examined with a UVP detection system and quantified with LABWORKS 4.6. β‐Actin protein was set as the internal control.

### 2.13 Statistical analysis

The data were expressed as mean ± SEM. Statistical analysis was performed via one‐way ANOVA and then using a Student's *t* test (SPSS Program, version 11.5; SPSS Inc., Chicago, IL, USA). Differences amongst groups were considered to be significant at *P* < 0.05.

## 3 RESULTS

### 3.1 Identification of triterpenoid saponins in the TSS by HPLC‐ESI‐MS/MS

**Figure 1:**
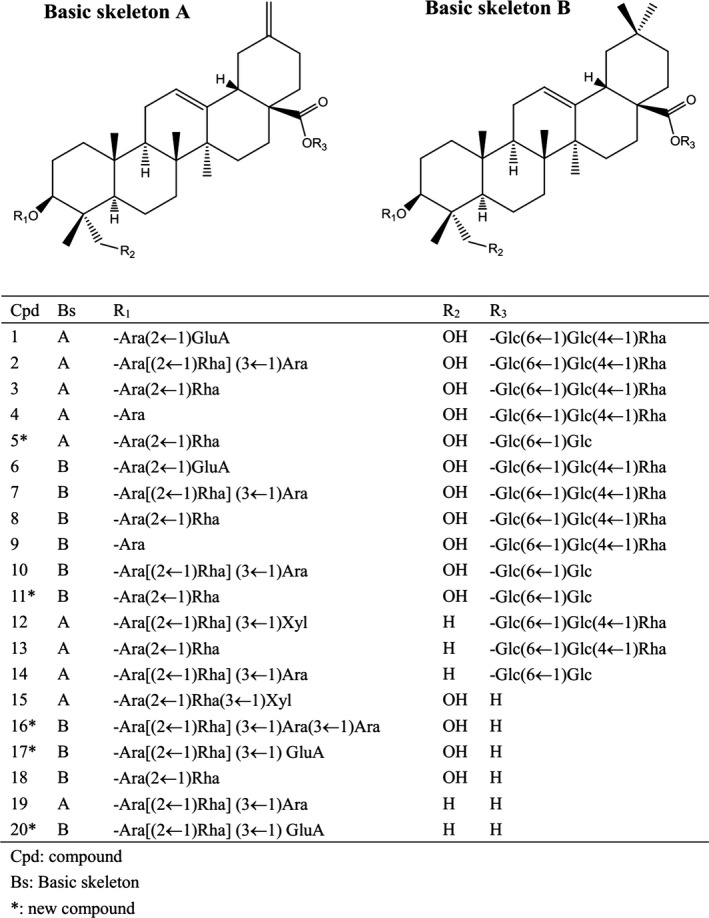
Chemical structures of triterpene saponins identified in TSS

Under the current chromatographic and MS conditions, almost 36 peaks were identified from the TSS. Twenty of these major peaks were established via contrasting the molecular formulae and fragmentation patterns with documented data in the literature. The information related to the detected compounds is condensed and revealed in Table 1 and the chemical structures are shown in Figure 1. Based on their sapogenins, the distinguished triterpene saponins can be separated into two basic types: 30‐nor‐oleanane‐type oligoglycosides (type A) or oleanane‐type oligoglycosides (type B). Compound 7 was used an example to discuss the structure elucidation in detail. Compound 7, detected at an RT of 16.3 minutes in TSS, displayed an 1397 [M+HCOO]^−^ ion at *m*/*z* 1397 and [M+Na]^+^ ion at 1375, indicating a molecular formula of C_64_H_104_O_30_. The MS^2^ spectrum of the ion at *m/z* 1375 [M+Na]^+^gave positive fragments at *m/z* 905 [M+Na‐470]^+^, 861 [M+Na‐470‐44]^+^ and 493 [470+Na]^+^, suggesting that there was a sugar chain rhamnopyranosyl‐(1→4)‐glucopyranosyl‐(1→6)‐glucopyranosyl connected to 7 by an ester bond. Other fragment ions at 729 [(M+Na‐470‐44)‐132]^+^, 583 [(M+Na‐470‐44)‐132‐146]^+^ and 451 [(M+Na‐470‐44)‐132‐146‐132]^+^ suggested the presence of another sugar chain ether‐linked in 7 comprising two pentoses and one deoxyhexose, in which two pentoses were at the terminal and inner position. The aglycone was determined to be 30‐norhederagenin. Therefore, the structure of 7 was tentatively assigned as yemuoside YM_32_ by comparison of the fragmentation patterns with a previous report.^15^ In the same way, the structures of the other triterpene saponins were determined as depicted in Figure 1.

**Table 1:** Triterpene saponins identified in the TSS by HPLC‐ESI‐MS/MS

No.	*t*_R_ (min)	Molecular formula	ESI‐MS	Identification	Refs.
1	7.7	C_58_H_90_O_28_	1257 [M + Na]^+^	Stauntoside K	39
2	9.2	C_63_H_100_O_30_	1359 [M + Na]^+^	Yemuoside YM_28_	15
3	9.8	C_58_H_92_O_26_	1249 [M+HCOO]^−^ 1227 [M + Na]^+^	Glycoside L‐H3	39
4	10.2	C_52_H_82_O_22_	1103 [M+HCOO]^−^ 1081 [M + Na]^+^	Glycoside L‐G1	40
5	12.3	C_52_H_82_O_22_	1103 [M+HCOO]^−^ 1081 [M + Na]^+^	New	NF
6	13.6	C_59_H_94_O_28_	1249 [M ‐ H]^−^ 1273 [M + Na]^+^	Stauntoside L	39
7	16.3	C_64_H_104_O_30_	1397 [M+HCOO]^−^ 1375 [M + Na]^+^	Yemuoside YM_32_	15
8	17.9	C_59_H_96_O_26_	1265 [M+HCOO]^−^ 1243 [M + Na]^+^	Hederasaponin C	39
9	19.3	C_53_H_86_O_22_	1119 [M+HCOO]^−^ 1097 [M + Na]^+^	Hederasaponin D	39
10	22.7	C_58_H_94_O_26_	1251 [M+HCOO]^−^ 1229 [M + Na]^+^	Yemuoside YM_35_	15
11	23.9	C_53_H_86_O_22_	1119 [M+HCOO]^−^ 1097 [M + Na]^+^	Dipsacoside B	41
12	27.4	C_63_H_100_O_29_	1365 [M+HCOO]^−^ 1343 [M + Na]^+^	Yemuoside YM_21_	42
13	31.1	C_58_H_92_O_25_	1233 [M+HCOO]^−^ 1211 [M + Na]^+^	Yemuoside YM_10_	42
14	33.2	C_57_H_90_O_25_	1219 [M+HCOO]^−^ 1197 [M + Na]^+^	Yemuoside YM_24_	42
15	45.0	C_45_H_70_O_16_	911 [M+HCOO]^−^ 889 [M + Na]^+^	Yemuoside YM_37_	43
16	48.9	C_51_H_82_O_20_	1059 [M+HCOO]^−^ 1037 [M + Na]^+^	New	NF
17	50.4	C_47_H_74_O_18_	925 [M‐H]^−^ 949 [M + Na]^+^	New	NF
18	53.7	C_41_H_66_O_12_	795 [M+HCOO]^−^ 751 [M + H]^+^	3‐*O*‐α‐l‐rhamnopyranosyl‐(1→2)‐α‐l‐arabinopyranosyl‐hederagenin	43
19	55.7	C_45_H_70_O_15_	849 [M‐H]^−^ 873 [M + Na]^+^	3‐*O*‐α‐l‐arabinopyranosyl‐(1→3)‐α‐l‐rhamnopyranosyl‐(1→2)‐α‐l‐arabinopyranosyl‐akebonic acid	43
20	60.0	C_47_H_74_O_17_	909 [M‐H]^−^ 933 [M + Na]^+^	3β‐[(*O*‐β‐d‐glucuronopyranosyl‐(1→3)‐*O*‐[α‐l‐rhamnopyranosyl‐(1→2)]‐α‐l‐arabinopyranosyl)oxy]olean‐12‐en‐28‐oic acid	44

NF: not found.

### 3.2 Acute toxicity study

Toxicological evaluation is a crucial constituent part of the drug development procedure.^19^ In the acute toxicity study, no deaths or bad clinical signs, such as no appetite, agitation, or uneasiness, was observed with the maximum dose of 2000 mg/kg. The results of the acute toxicity study indicated that the TSS was safe up to a single dose of 2000 mg/kg BW. Coupled with the fact that the type 2 diabetic rats showed an anti‐diabetic effect at the dose of saponins at 50‐200 mg/kg,^20^ the concentration of TSS for further analysis was fixed as 30, 60 and 120 mg/kg.

### 3.3 Effect on BW

**Figure 2:**
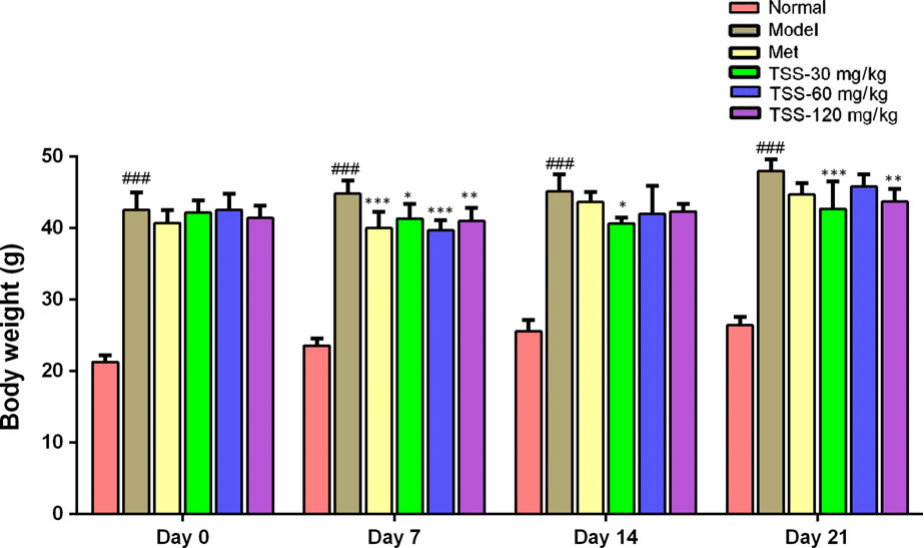
Effects of total saponins on body weight in type 2 diabetic *db/db* mice (n = 6). ^###^ *P* < 0.001 in contrast to the normal group; *P < 0.05 in contrast to the model group; **P < 0.01 in contrast to the model group; and ***P < 0.001 in contrast to the model group

Obesity is a critical risk factor for T2DM, and the regulation of BW has a large impact on improving insulin sensitivity.^21^ At the end of the study, the type 2 diabetes *db/db* mice showed a 12.75% increase in BW compared to the first day. Treatment with metformin resulted in a 9.82% increase in BW. Following 21 days of TSS treatment at 30, 60 and 120 mg/kg, BW was increased by 3.10%, 7.66% and 3.67%, respectively (Figure 2). TSS reduced the rate of weight increase in contrast to the *db/db* group.

### 3.4 Effects on FBG, OGTT, ITT and serum insulin levels

**Figure 3:**
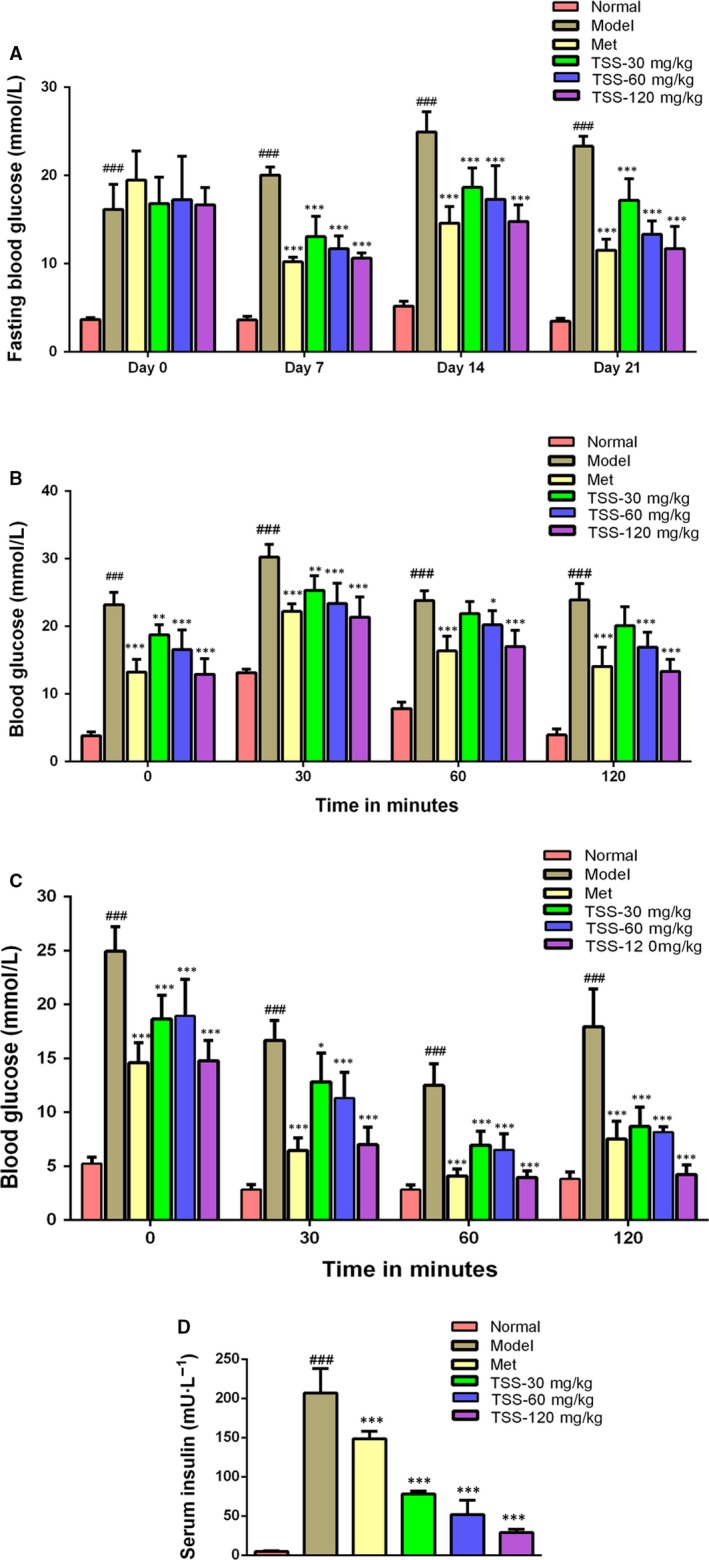
Effects of total saponins on fasting blood glucose levels (A), oral glucose tolerance (OGTT) (B), insulin tolerance (ITT) (C) and serum insulin content (D) in type 2 diabetic *db/db* mice (n = 6). ^###^ *P* < 0.001 in contrast to the normal group; *P < 0.05 in contrast to the model group; **P < 0.01 in contrast to the model group and ***P < 0.001 in contrast to the model group

The alterations in the FBG level of the mice are shown in Figure 3A. FBG levels of the *db/db* mice were significantly elevated, which was in contrast to the normal control group (*P* < 0.001). Metformin (200 mg/kg) caused a significant reduction in FBG levels in contrast to the *db/db* mice (*P* < 0.001). Each of the three doses of TSS significantly inhibited the increase in FBG levels in *db/db* mice throughout the entire experiment (*P* < 0.001) and showed dose‐dependent manners. Following the TSS treatment at 120, 60 and 30 mg/kg for 7 days, the FBG level was reduced by 36.33%, 32.20% and 22.37%, respectively. The model and metformin‐treated (200 mg/kg) mice showed a 24.05% increase and a 47.54% decrease in FBG, respectively. On day 14, the FBG values of the three TSS groups only increased slightly compared to day 7. Upon the conclusion of the experiment (day 21), the FBG levels of the three TSS groups 30, 60 and 120 mg/kg were reduced by 2.18%, 22.74% and 29.72%, respectively, in contrast with the FBG levels at day 0, whilst the FBG levels of the model group were elevated by 44.31%. The FBG levels of two TSS groups 60 and 120 mg/kg were similar to the metformin (200 mg/kg) group (*P* > 0.05).

The OGTT results are shown in Figure 3B. The TSS treatment at 30, 60, 120 mg/kg revealed a highly significant lowering of plasma glucose levels. At 30 minutes following glucose administration, the blood glucose levels were reduced at 120 mg/kg compared with the metformin group. The ITT results are shown in Figure 3C. At 30 minutes following an insulin injection, the diabetic model, the metformin‐treated mice, and the 120 mg/kg TSS‐treated showed a 33.09%, 55.83% and 52.64% reduction in plasma glucose, respectively. At 60 minutes following an insulin injection, the diabetic model, the metformin‐treated mice and the 120 mg/kg TSS‐treated mice showed a 49.87%, 73.17% and 71.92% reduction in plasma glucose levels, respectively. TSS treatment at a dose of 120 mg/kg significantly enhanced the insulin‐induced decrease in plasma glucose levels.

As revealed in Figure 3D, the serum insulin levels in the *db/db* mice were significantly elevated compared to the normal mice (*P* < 0.001). Following 3 weeks of treatment with 30, 60, 120 mg/kg TSS and 200 mg/kg metformin, the serum insulin levels were decreased by 62.22%, 74.88%, 86.08% and 28.20% in contrast to those of the *db/db* model mice.

### 3.5 Effects on liver and skeletal muscle glycogen levels

**Figure 4:**
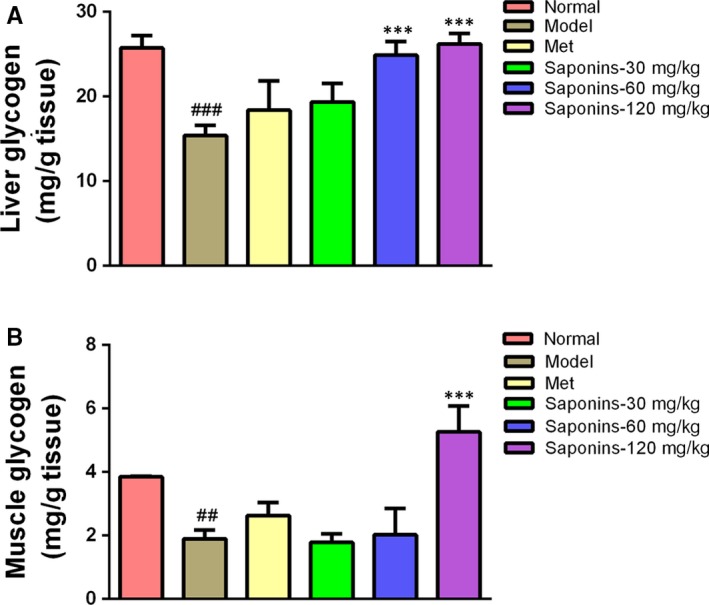
Effects of total saponins on liver glycogen levels (A) and muscle glycogen levels (B) in type 2 diabetic *db/db* mice (n = 6). ^##^ *P* < 0.01 in contrast to the normal group; ^###^ *P* < 0.001 in contrast to the normal group; and ***P < 0.001 in contrast to the model group

The liver glycogen levels in the diabetic model mice were noticeably reduced compared to that of the normal mice (*P* < 0.001, Figure 4A). In contrast to the model mice, TSS treatments at doses of 60 and 120 mg/kg led to a significant increase in liver glycogen levels (*P* < 0.001, Figure 6A). Metformin could increase the liver glycogen level but this effect was not statistically significant. The muscle glycogen levels were also down‐regulated in the model mice when compared to the normal mice (*P* < 0.01, Figure 4B). Treatments using metformin and 120 mg/kg TSS, resulted in a significant increase in muscle glycogen levels compared to the model mice (*P* < 0.001, Figure 4B). The muscle glycogen levels of the 120 mg/kg TSS group (5.263 ± 0.812 mg/g tissue) were higher than that of the metformin group (2.631 ± 0.406 mg/g tissue).

### 3.6 Effects on serum lipid profile

The TC, TG, HDL‐C and LDL‐C of the *db/db* model mice were all upregulated significantly when compared to the normal mice (*P* < 0.001, Table 2). Compared with the *db/db* model mice, the serum TG levels were significantly reduced with the treatment of 30, 60, 120 mg/kg TSS and 200 mg/kg metformin (*P* < 0.001), respectively. The LDL‐C levels were reduced significantly with 120 mg/kg TSS (*P* < 0.01) and metformin (*P* < 0.001), respectively. The HDL‐C levels were elevated with 30, 60 and 120 mg/kg TSS and metformin, respectively. However, the increase in HDL‐C levels with the treatment of 30 and 60 mg/kg TSS and metformin were not statistically significant.

**Table 2:** Effect of TSS on serum lipid levels in T2DM *db/db* mice (n = 6)

Group	TC (mmol/L)	TG (mmol/L)	HDL‐C (mmol/L)	LDL‐C (mmol/L)
Normal	2.506 ± 0.154	0.874 ± 0.112	1.503 ± 0.051	0.229 ± 0.023
Model	4.203 ± 0.326###	2.512 ± 0.674###	2.205 ± 0.078###	0.306 ± 0.030##
Met	3.847 ± 0.414	1.165 ± 0.135***	2.608 ± 0.330	0.190 ± 0.045***
Saponins‐30 mg/kg	3.467 ± 0.366*	1.467 ± 0.106***	2.108 ± 0.166	0.265 ± 0.051
Saponins‐60 mg/kg	3.737 ± 0.633	1.363 ± 0.127***	2.260 ± 0.402	0.253 ± 0.035
Saponins‐120 mg/kg	3.832 ± 0.257	1.056 ± 0.156***	2.680 ± 0.160*	0.223 ± 0.020**

Values are mean ± SEM, n = 6. ^##^ *P* < 0.01 in contrast to the normal group; ^###^ *P* < 0.001 in contrast to the normal group; *P < 0.05 in contrast to the model group; **P < 0.01 in contrast to the model group; ***P < 0.001 in contrast to the model group.

### 3.7 Effects on pathological changes of the liver

**Figure 5:**
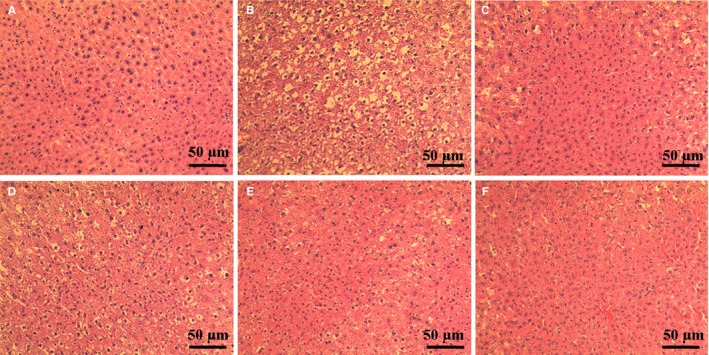
Effects of total saponins on pathological changes in the liver of type 2 diabtetic *db/db* mice (n = 3) (HE staining, magnification: ×200). (A) The normal mice; (B) type 2 diabetes model *db/db* group; (C) 200 mg/kg metformin group; (D) 30 mg/kg TSS group; (E) 60 mg/kg TSS group; (F) 120 mg/kg TSS group

The pathological changes in the liver were shown in Figure 5. The livers of *db/db* model mice showed macrovesicular cell, sinusoid dilation and hepatocellular degeneration, reflecting the hepatic steatosis. The TSS treatments decreased the hepatic steatosis in a dose‐dependent manner.

### 3.8 Effects on the insulin receptor substrates‐1/PI3K/AKT pathway in liver tissues

**Figure 6:**
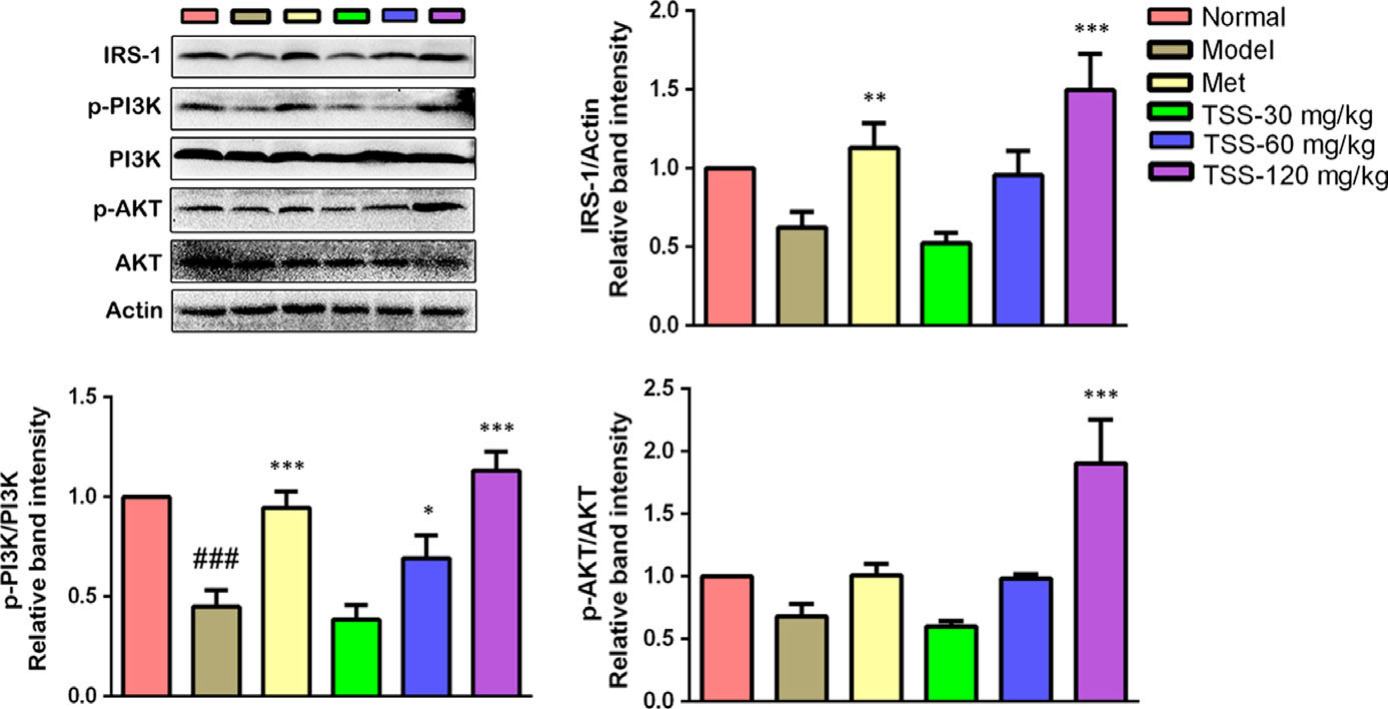
Effects of total saponins on the IRS‐1/PI3K/AKT pathway in liver tissues of type 2 diabetic *db/db* mice (n = 3). The protein expressions are shown as the mean grey scale of the matching bands in the bar graph. ^###^ *P* < 0.001 in contrast to the normal group; *P < 0.05 in contrast to the model group; **P < 0.01 in contrast to the model group; and ***P < 0.001 in contrast to the model group

To examine the mechanism in charge of the saponin‐stimulated glucose uptake, the effects of TSS on the insulin receptor substrates (IRS)‐1/PI3K/AKT pathway were evaluated. The Western blotting revealed that TSS elevated the expression of IRS‐1 and stimulated the phosphorylation of PI3K and AKT in a dose‐dependent manner. The administration of 120 mg/kg TSS to the *db/db* mice resulted in a significant increase in the levels of IRS‐1, p‐PI3K ‐p85α Tyr^508^ and p‐AKT Ser^473^ levels in the liver tissue in contrast to those in the *db/db* mice (*P* < 0.001) (Figure 6).

### 3.9 Effects on glucose transporter 4 levels in skeletal muscles

**Figure 7:**
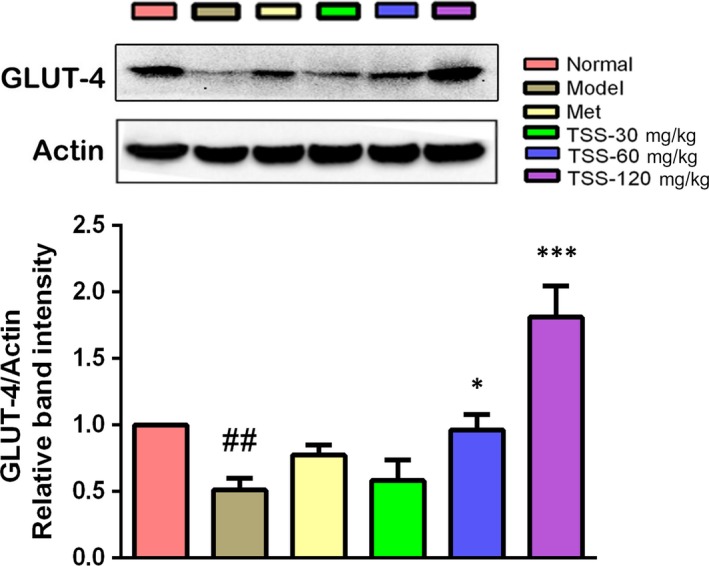
Effects of total saponins on GLUT4 protein levels in muscle tissues of type 2 diabetic *db/db* mice (n = 3). The protein expressions are shown as the mean grey scale of the matching bands in the bar graph. ^##^ *P* < 0.01 in contrast to the normal group; *P < 0.05 in contrast to the model group; and ***P < 0.001 in contrast to the model group

In skeletal muscle, glucose uptake is primarily credited with improved translocation and expression of glucose transporter 4 (GLUT4),^22^ so GLUT4 expression in skeletal muscle was determined by Western blotting analysis. GLUT4 levels decreased significantly in the *db/db* mice compared to the normal mice (*P* < 0.01). Metformin also induced an increase in GLUT4 expression; however, this was not statistically significant. TSS caused a dose‐dependent increase in the expression levels of GLUT4. The 60 mg/kg (*P* < 0.05) and 120 mg/kg (*P* < 0.001) TSS treatments could significantly upregulate the expression of GLUT4 protein, respectively (Figure 7).

### 3.10 Effects on the AMPK and ACC in liver tissues

**Figure 8:**
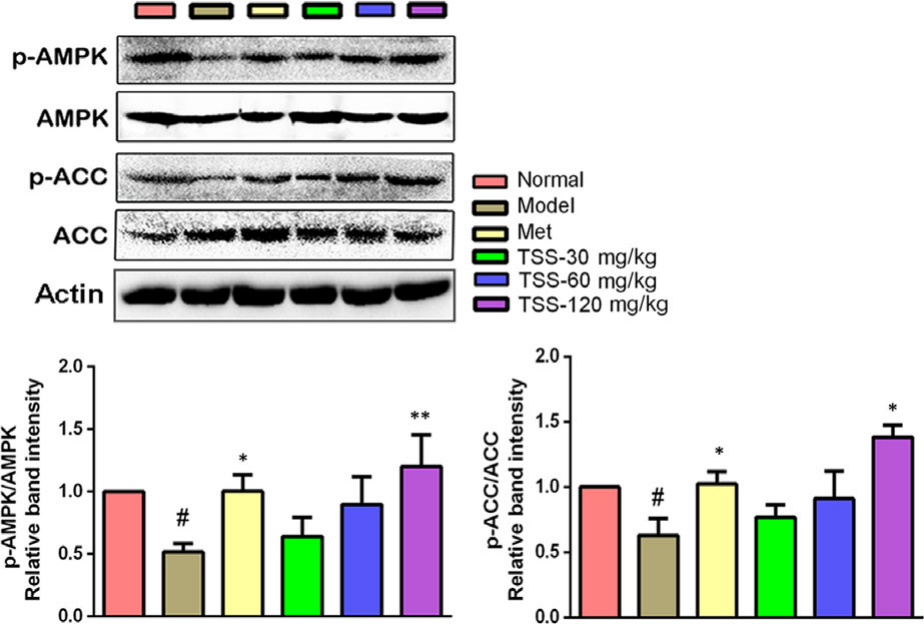
Effects of total saponins on phosphorylated AMPK and ACC in liver tissues of type 2 diabetic *db/db* mice (n = 3). The protein expressions are shown as the mean grey scale of the matching bands in the bar graph. ^#^ *P* < 0.05 in contrast to the normal group; *P < 0.05 in contrast to the model group; and **P < 0.01 in contrast to the model group

Due to the importance of phosphorylated AMPK and ACC in lipid metabolism and the anti‐hyperlipidemia effects of TSS, protein levels of phosphorylated AMPK and ACC were examined by western blotting. Metformin treatment in the *db/db* mice resulted in a significant increase in p‐AMPK Thr^172^ levels (*P* < 0.05). The administration of TSS elevated the p‐AMPK Thr^172^ levels in a dose‐dependent manner in the *db/db* mice. Treatments with 120 mg/kg TSS significantly activated the phosphorylation of AMPK Thr^172^ (*P* < 0.01). Meanwhile, metformin and 120 mg/kg TSS increased the phosphorylation of ACC Ser^79^ in the *db/db* mice, respectively (*P* < 0.05) (Figure 8).

## 4 DISCUSSION

T2DM is distinguished by hyperglycemia, which is very dangerous for diabetics.^23^ Hyperglycemia damages the prooxidant/antioxidant balance, lowers antioxidant levels and elevates the levels of free radicals, which then destroy pancreatic β‐cells and induce insulin resistance.^24^ Our investigation revealed that the TSS significantly decreases FBG, OGTT and ITT levels in the *db/db* mice (Figure 3), which is in agreement with the results of saponins from other plants. Saponins from the seed of *Entada phaseoloides* significantly lowered FBG, serum insulin levels and reduced hyperglycemia in T2DM rats.^11^ In addition, the saponins from the root of *Panax notoginseng* lowered FBG levels and revealed anti‐hyperglycemic effects on KK‐Ay mice.^20^, ^25^ These results demonstrated that saponins could improve glucose homeostasis and insulin resistance. The ability of TSS to reducing hyperglycemia makes TSS an excellent candidate for the treatment of T2DM.

Glucose homeostasis is primarily controlled by the liver and skeletal muscles, where the majority of glucose is desposited as glycogen. The liver is central to glucose homeostasis by the conversion of glucose into glycogen in the postprandial state and by the conversion of glycogenolysis into glucose in the fasting state.^26^ The glycogen levels in the muscles and liver are an immediate reflection of insulin sensitivity because insulin encourages intracellular glycogen deposition via a coordinated elevation in glucose movement and glycogen synthesis.^12^ Insulin resistance is distinguished by damage in muscle glucose uptake and the overproduction of glucose in the liver. The liver and muscle glycogen content is significantly lower in diabetic animals,^27^ which is in proportion to insulin deficiency.^28^ TSS raised the glycogen levels in the liver and muscles of the *db/db* mice to a normal level, which could be because of elevated sensitivity to insulin.

PI3K/AKT pathway activation is required to modulate IRS. Of the IRS, IRS‐1 is closely connected to glucose homeostasis in the liver.^4^ The phosphorylation of IRS‐1 activates PI3K, which causes the production of phosphatidylinositol 3,4,5‐trisphosphate, leading to the activation of AKT.^29^ AKT is a one of the most critical and flexible protein kinases and it modulates nutrient uptake and metabolism in a cell‐intrinsic fashion via various downstream targets. One of the most pivotal physiological functions of AKT is to precisely control the biological actions of insulin in glucose metabolism.^30^ AKT activation can prompt glycogen synthesis and improve the rate of glycolysis.^31^ Western blotting analysis revealed that TSS activated the proteins in the IRS‐1/PI3K/AKT signal pathway in the *db/db* mice (Figure 6). These findings suggested that the activation of the IRS‐1/PI3K/AKT insulin signalling cascade is in charge of the pharmacological activity of TSS on hepatic glycogen synthesis.

GLUT4, a 12 transmembrane domain protein, plays a pivotal part in glucose homeostasis through its translocation and expression in muscle.^22^ Insulin induces GLUT4 into the plasma membrane, and this translocation from the intracellular areas seems to fail because of the insulin resistance in T2DM.^32^ GLUT4 overexpression exclusively in the skeletal muscle increases whole‐body insulin activity and glucose homeostasis.^33^ Therefore, raising GLUT4 expression in skeletal muscles may be an efficient therapy for T2DM.^34^ Western blotting analysis revealed that the GLUT4 expression levels in the skeletal muscles of *db/db* mice was significantly raised by TSS. The activation of AKT causes GLUT4 translocation to the plasma membrane, which in turn enhances elevate glucose uptake.^35^ The present study has revealed that TSS stimulates the AKT signalling pathway, we concluded that TSS could have a hypoglycemic impact in part because of AKT‐dependent stimulation of glucose uptake into skeletal muscles via GLUT4 expression.

Lipid metabolism disorder is always connected to the development of insulin resistance and T2DM, and hyperlipidemia is the main factor that causes cardiovascular diseases.^22^ Hypertriglyceridemia, hypercholesterolemia and reduced levels of high‐density lipoprotein are the most frequently diagnosed lipid abnormalities in T2DM.^36^ Following TSS treatment, the TG and LDL‐C levels in the *db/db* mice decreased, whilst HDL‐C levels increased (Table 2), suggesting that TSS could control hyperlipidemia and may substantially lower the risk of cardiovascular disease in T2DM patients. TSS‐regulated hyperlipidemia could be related to the regulation of the expression of various genes that are relevant to in lipid metabolism and transport.^9^

AMPK plays pivotal part in regulating lipid metabolism by controlling downstream ACC.^4^ The phosphorylation of Thr‐172, the primary stimulatory phosphorylation site of the α subunit, is necessary for AMPK activity. It has been documented that metformin enhances AMPK phosphorylation at Thr‐172,^37^ which agrees with the results of the present study (Figure 8). Meanwhile, high‐dose TSS induced AMPKα phosphorylation at Thr‐172 and this effect was more intensive than that of metformin (Figure 8). ACC, a required downstream effector in the AMPK signalling pathway, has a crucial part in hepatic lipid metabolism via catalysing the biosynthesis of malonyl‐CoA, which acts as the first substrate for fatty acid biosynthesis and as a strong inhibitor of carnitine palmitoyltransferase I, the rate‐limiting step for mitochondrial fatty acid oxidation.^38^ AMPK halts ACC via phosphorylation of Ser‐79, which is in agreement with the elevation in ACC phosphorylation at Ser‐79 induced by metformin.^37^ ACC phosphorylation was also increased by TSS, suggesting a saponin‐activated AMPK signalling pathway.

**Figure 9:**
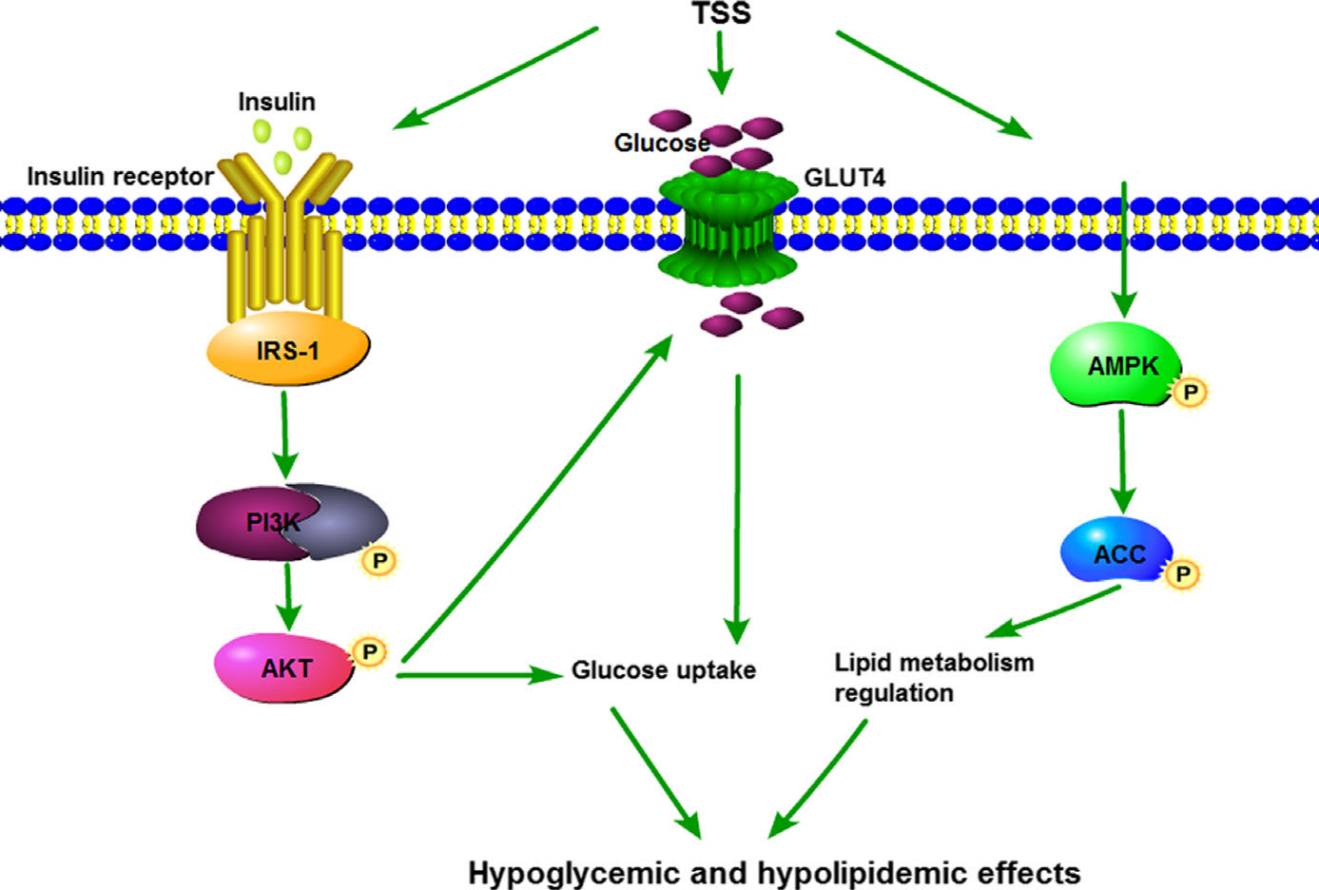
Proposed mechanism of TSS in the improvement in glycaemic and lipid metabolism in *db/db* mice. TSS stimulates the IRS‐1/PI3K/AKT and AMPK/ACC pathways and GLUT4 protein expression. The arrows indicate the possible targets of TSS

In conclusion, TSS was obtained by HP‐20 microresin column chromatography and their chemical composition was analysed by LC‐ESI‐MS/MS. A total of 36 triterpene saponins were identified in TSS. Of these, 20 triterpene saponins were determined by contrasting the molecular formulae and fragmentation patterns with documented data in previous reports. All of the identified triterpene saponins belonged to the oleanane‐type. TSS exhibited hypoglycemic activity by lowering blood glucose levels, reducing plasma insulin levels, restoration of insulin response, and stimulating glycogen synthesis in the *db/db* mice. Additionally, TSS could modulate hyperlipidemia associated with diabetes. The mechanism of the hypoglycemic activity of TSS was established via the regulation of the IRS‐1/PI3K/AKT signalling pathway and GLUT4 expression. The activation of the AMPK/ACC signalling pathway might be responsible for the hypolipidemia activity of TSS (Figure 9). This investigation provides the foundation for the development of saponins based on *S. chinensis* for the treatment of T2DM.

## CONFLICT OF INTEREST

The authors confirm that there are no conflicts of interest.
