# Supplementary material for: Hypoglycemic and hypolipidemic effects of total saponins from Stauntonia chinensis in diabetic db/db mice
Source: J Cell Mol Med. 2018 Oct 16;22(12):6026–38. doi: 10.1111/jcmm.13876 (PMC6237556; doi:10.1111/jcmm.13876)
Supplement: Supplementary file 1 [file JCMM-22-6026-s001.doc]

**Hypoglycemic and hypolipidemic effects of total saponins from *Stauntonia chinensis* in diabetic *db/db* mice**

Jing Xu^1^, Sha Wang^1^, Tianhui Feng^2^, Yu Chen^2^* and Guangzhong Yang^1,3^*

^1^ School of Pharmaceutical Sciences, South-Central University for Nationalities, Wuhan 430074,

P. R. China

^2^ College of Chemistry and Material Sciences, South-Central University for Nationalities, Wuhan 430074, P. R. China

^3^National Demonstration Center for Experimental Ethnopharmacology Education, South-Central University for Nationalities, Wuhan 430074, P. R. China

**Electronic Supplementary Information**

1. Figure 1: MS spectra of compound **1** page 3
2. Figure 2: MS spectra of compound **2** page 4
3. Figure 3: MS spectra of compound **3** page 5-6
4. Figure 4: MS spectra of compound **4** page 6-7
5. Figure 5: MS spectra of compound **5** page 8
6. Figure 6: MS spectra of compound **6** page 9-10
7. Figure 7: MS spectra of compound **7** page 11-12
8. Figure 8: MS spectra of compound **8** page 13
9. Figure 9: MS spectra of compound **9** page 14
10. Figure 10: MS spectra of compound **10** page 15
11. Figure 11: MS spectra of compound **11** page 16
12. Figure 12: MS spectra of compound **12** page 17-18
13. Figure 13: MS spectra of compound **13** page 19
14. Figure 14: MS spectra of compound **14** page 20
15. Figure 15: MS spectra of compound **15** page 21-22
16. Figure 16: MS spectra of compound **16** page 23
17. Figure 17: MS spectra of compound **17** page 24-25
18. Figure 18: MS spectra of compound **18** page 26
19. Figure 19: MS spectra of compound **19** page 27
20. Figure 20: MS spectra of compound **20** page 28-29
21. Figure 21: MS total ion current (TIC) chromatogram of TSS page 30
22. Table 1: Triterpenoid saponins identified in the TSS by HPLC-ESI-MS/MS page 31-35


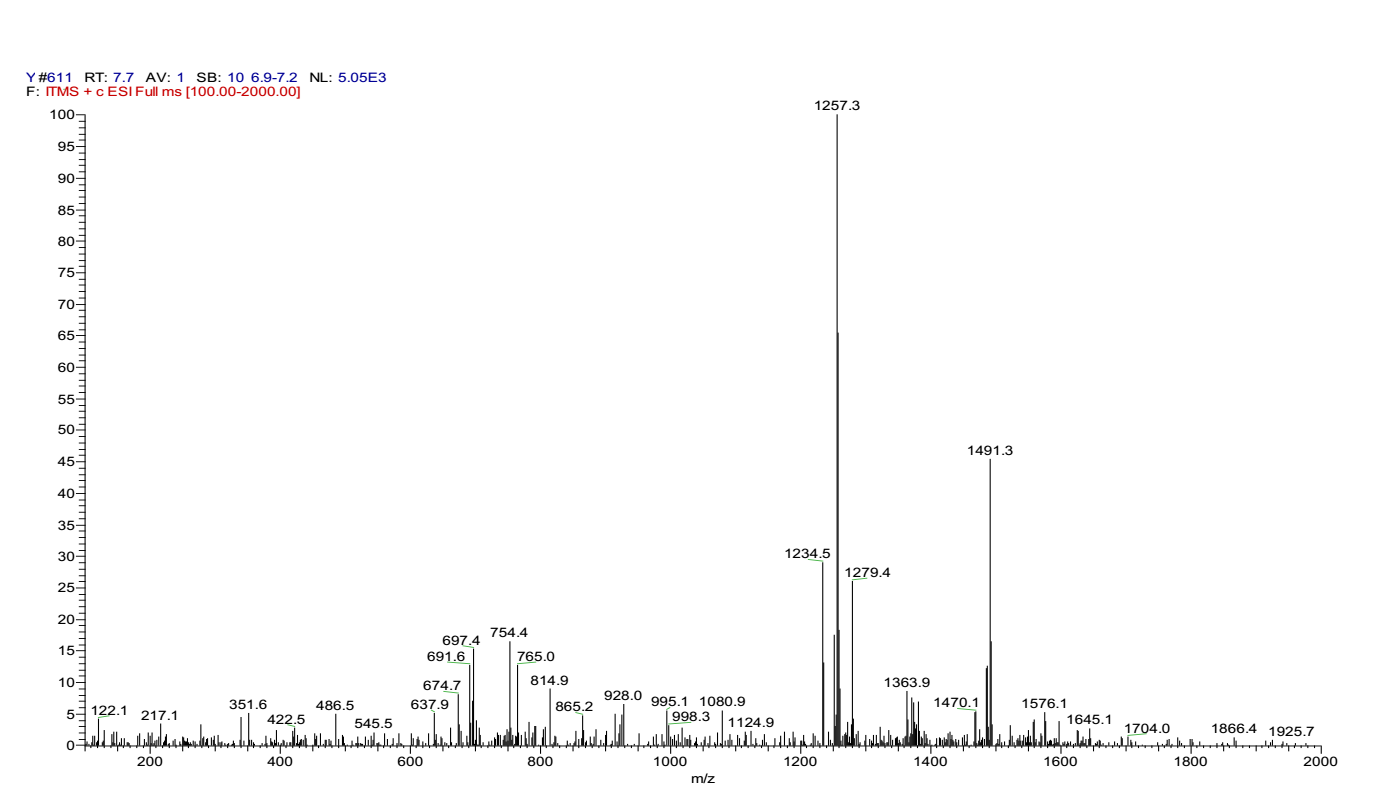


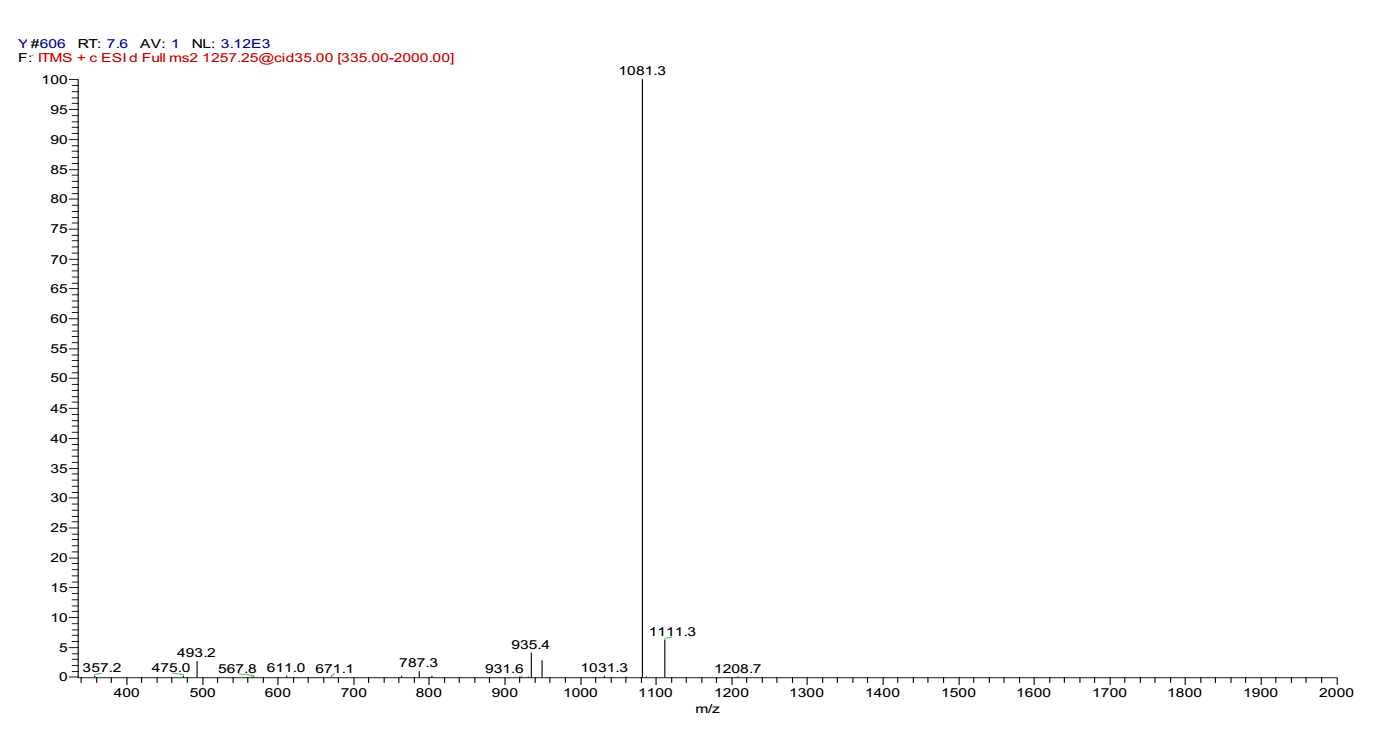


Figure 1: MS spectra of compound **1**


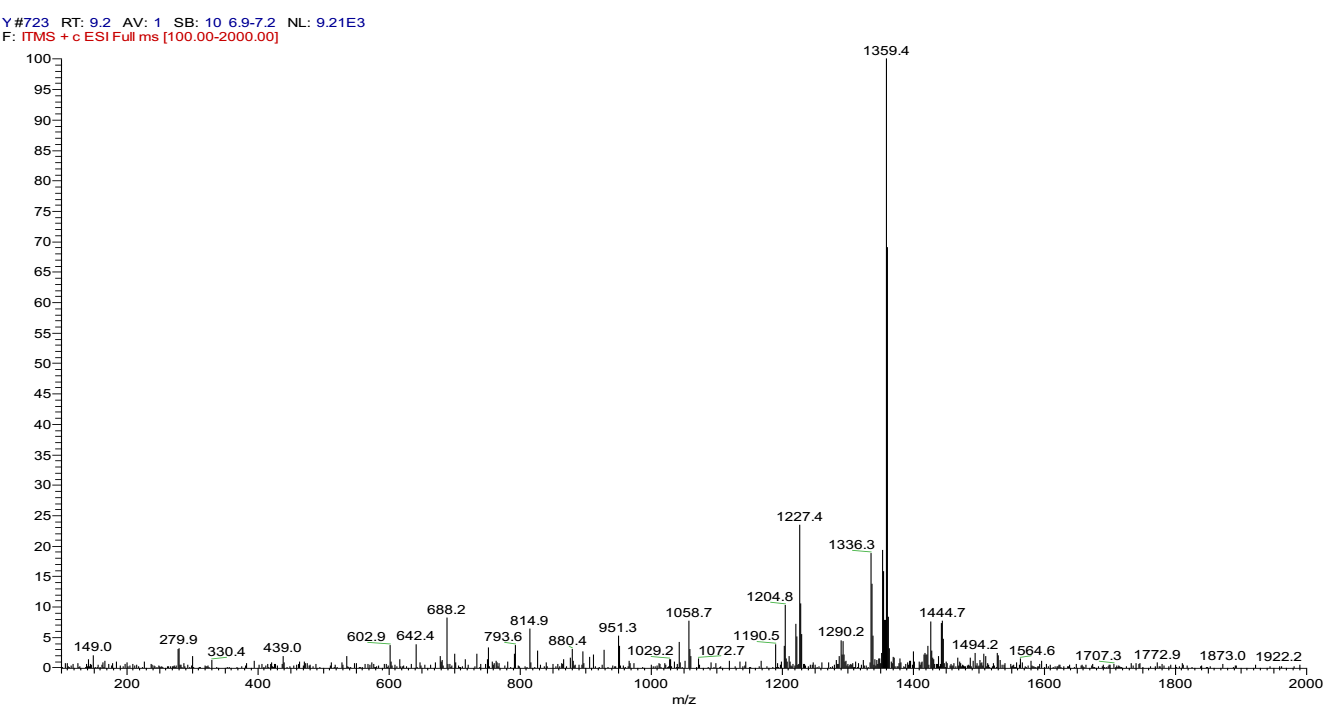


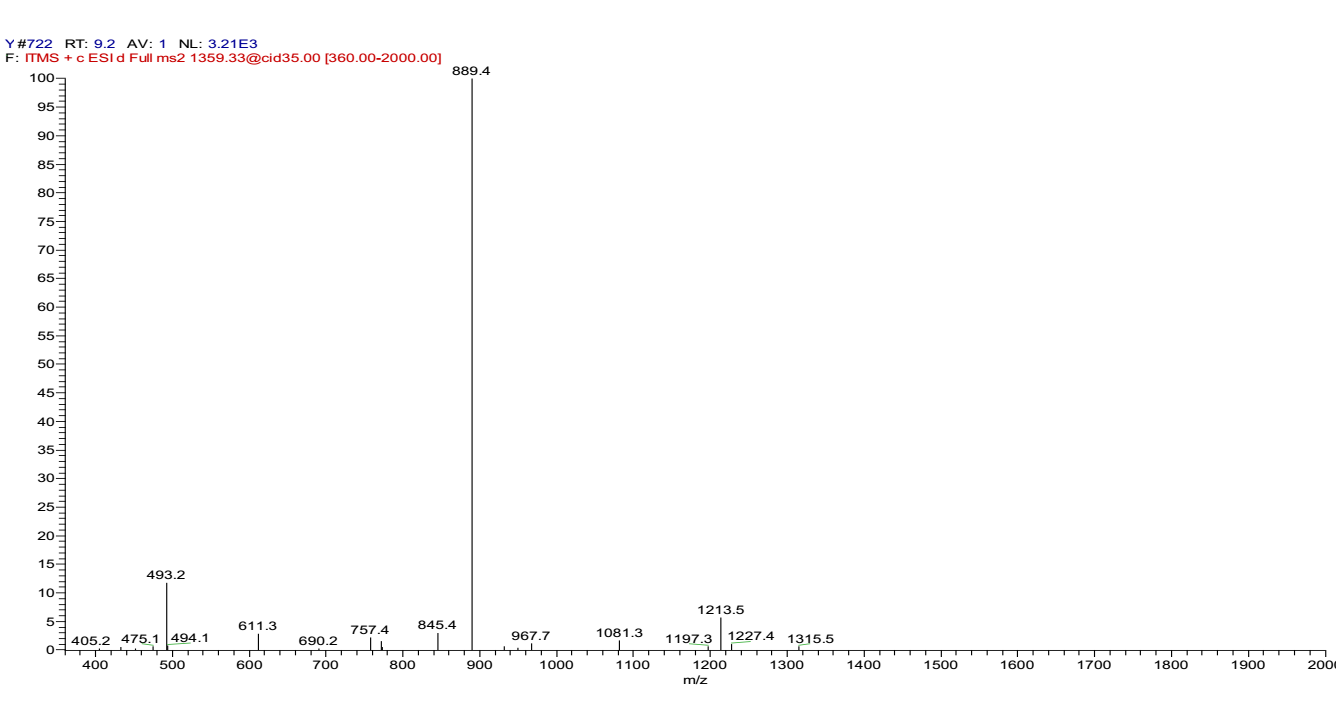


Figure 2: MS spectra of compound **2**


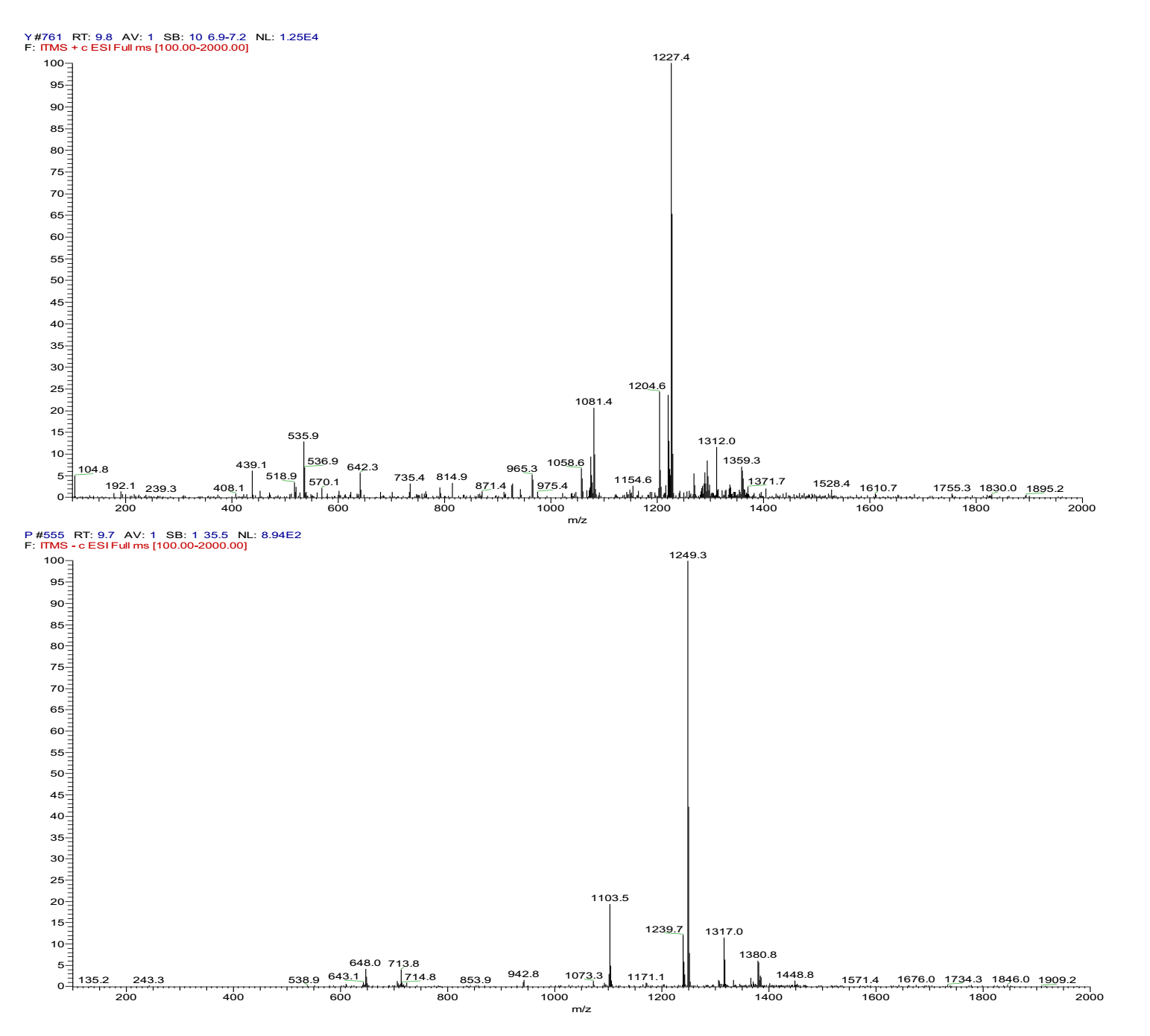


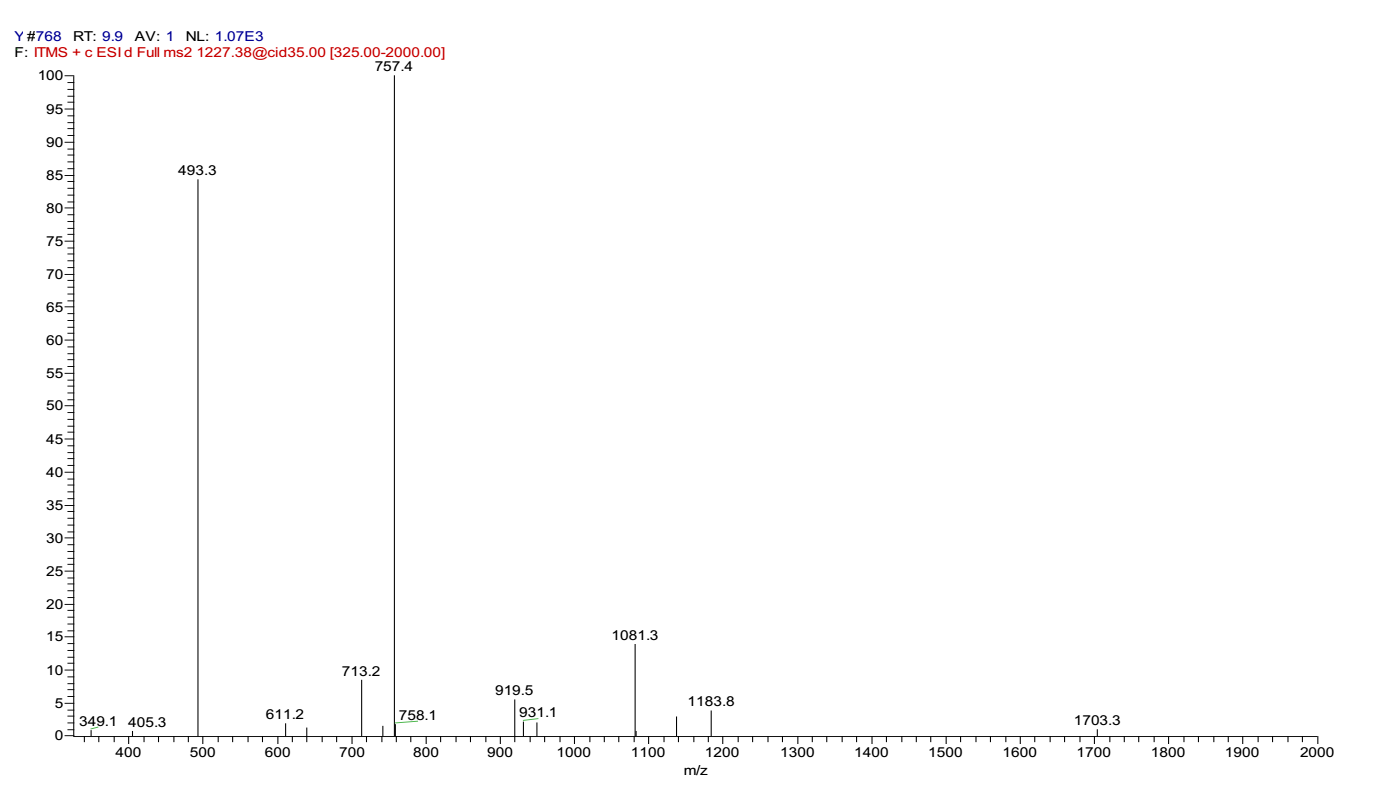


Figure 3: MS spectra of compound **3**


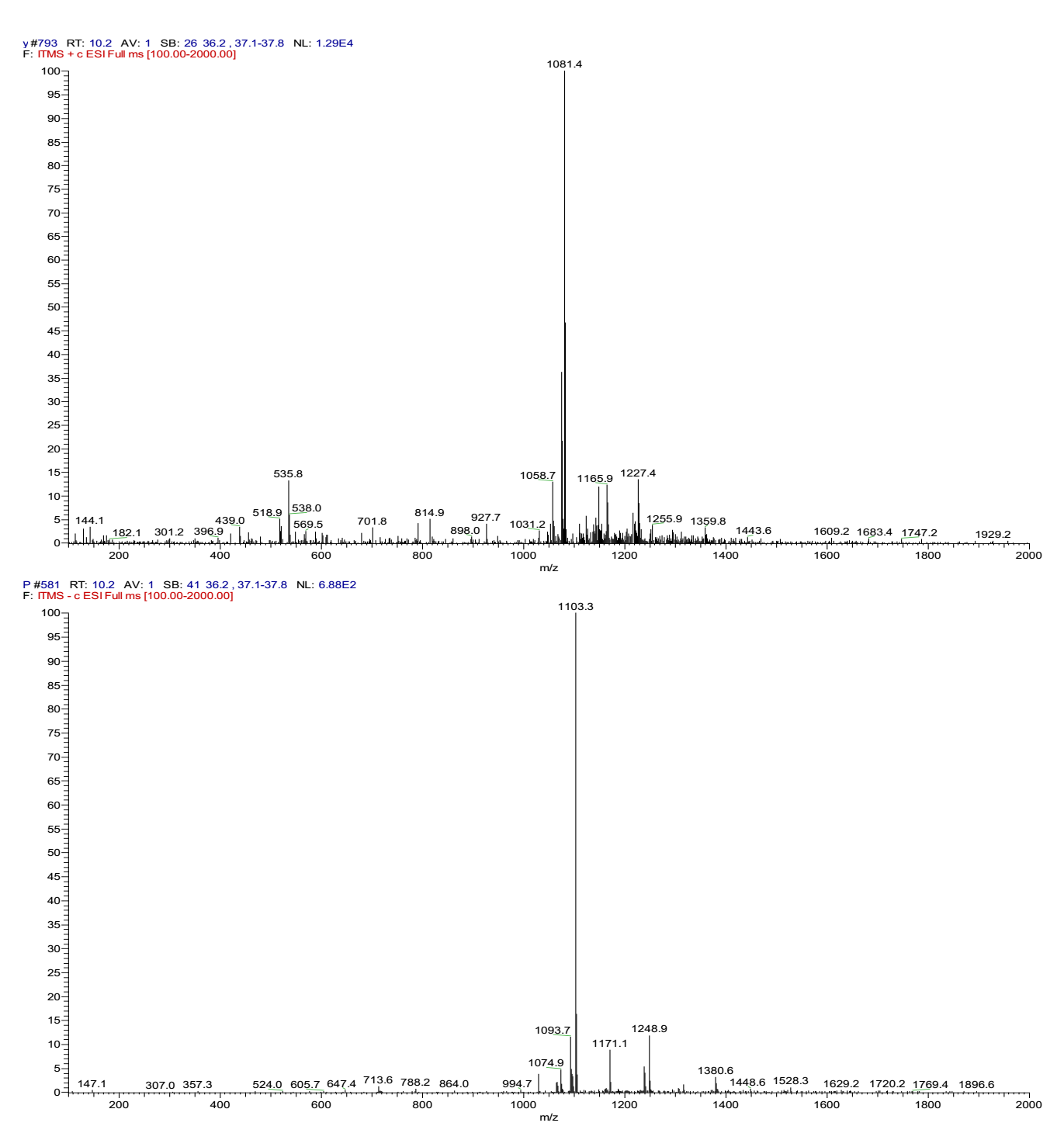


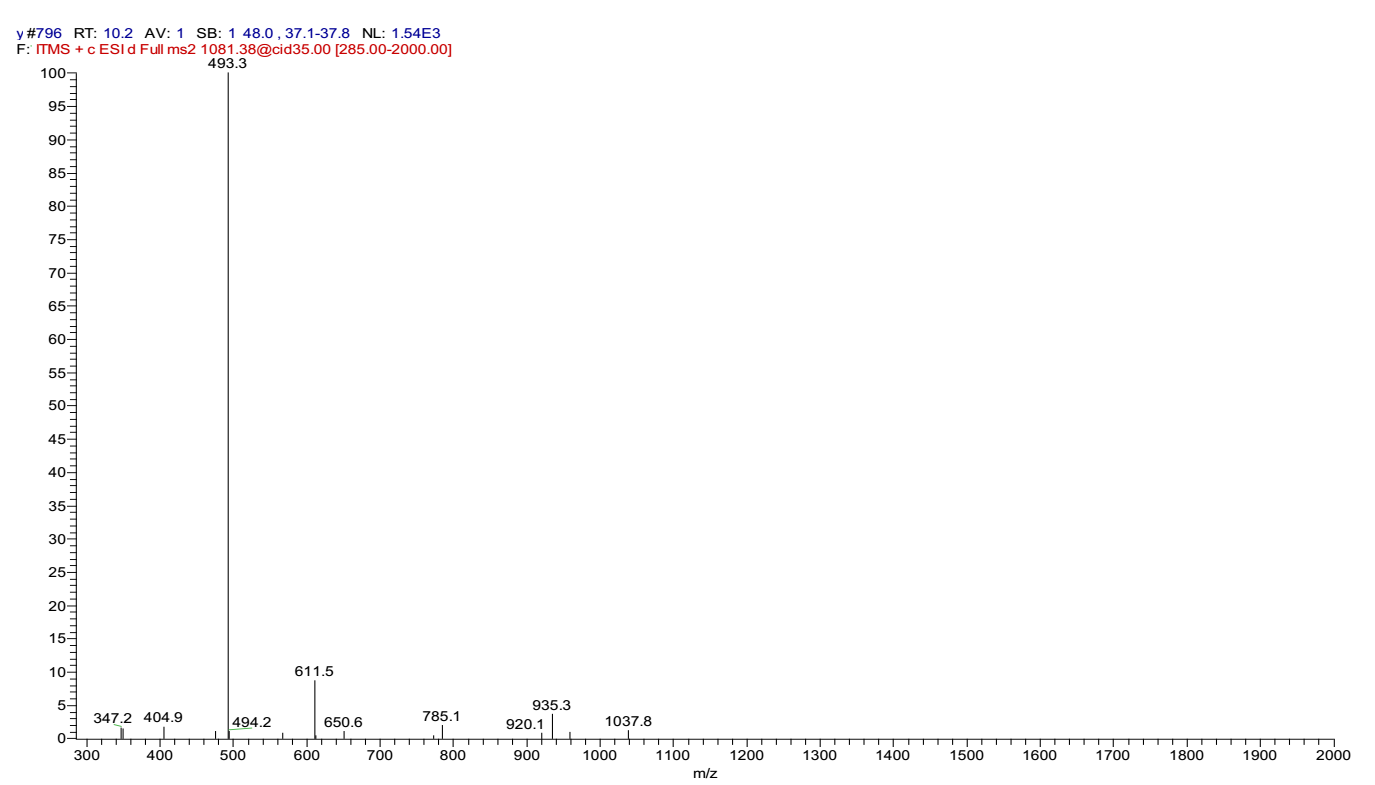


Figure 4: MS spectra of compound **4**


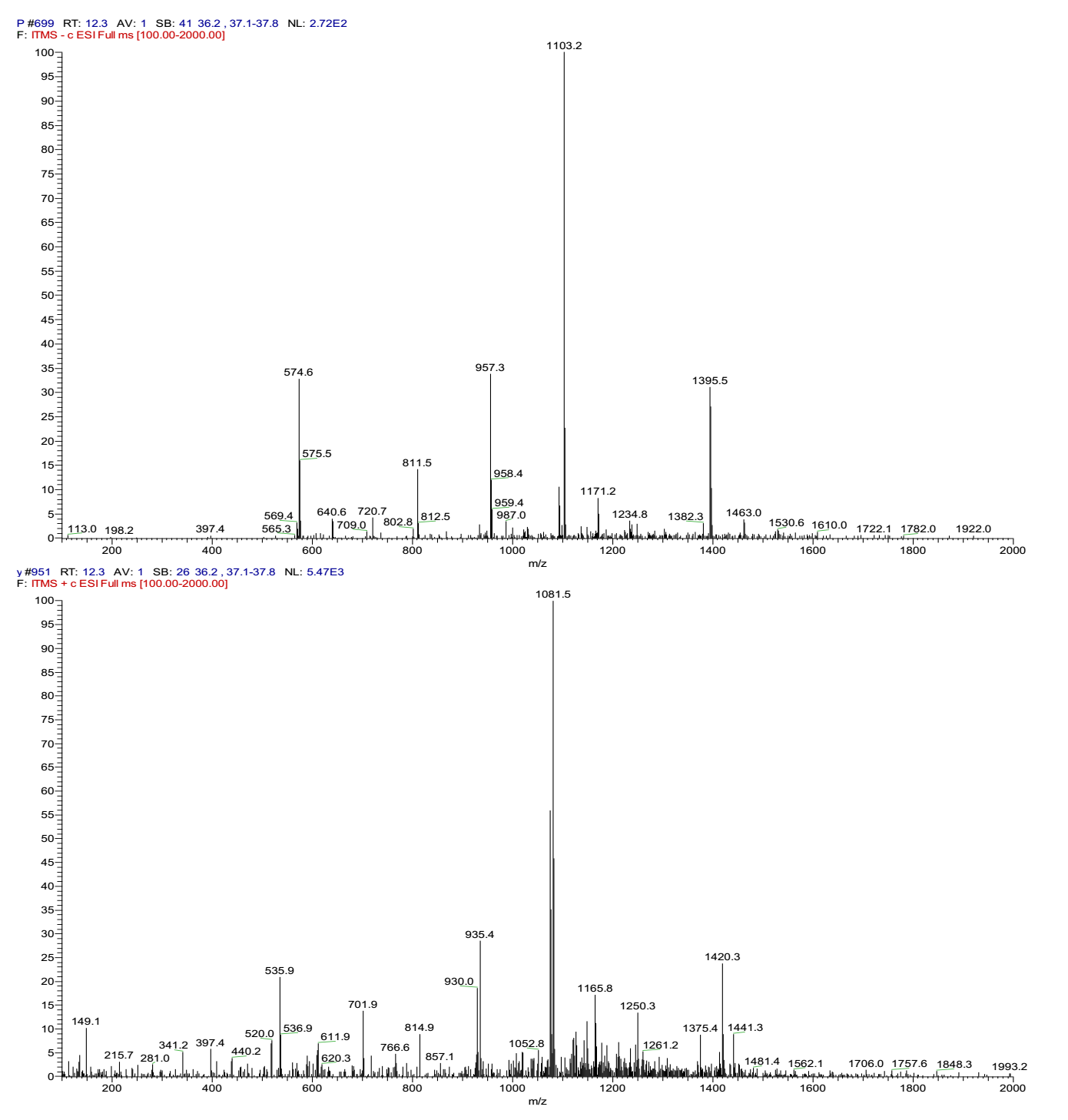


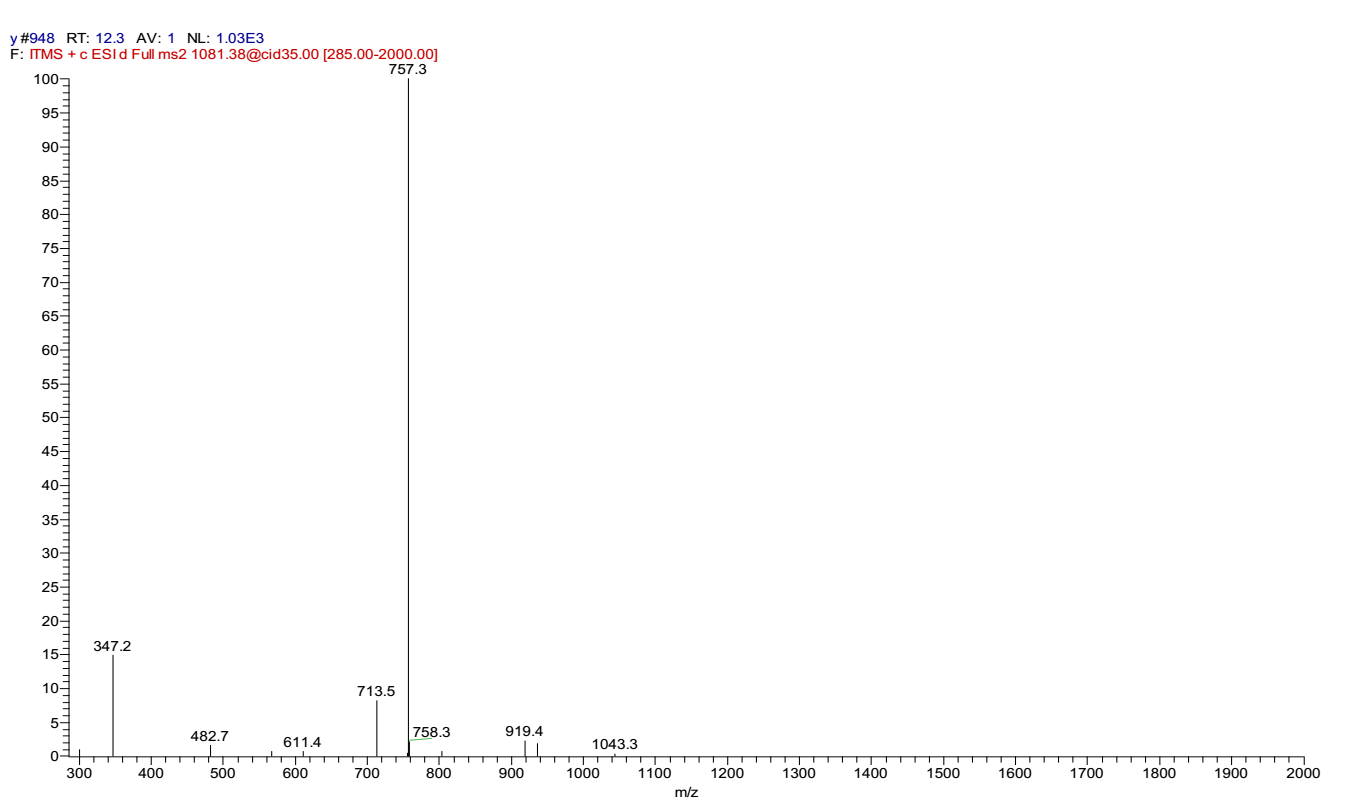


Figure 5: MS spectra of compound **5**


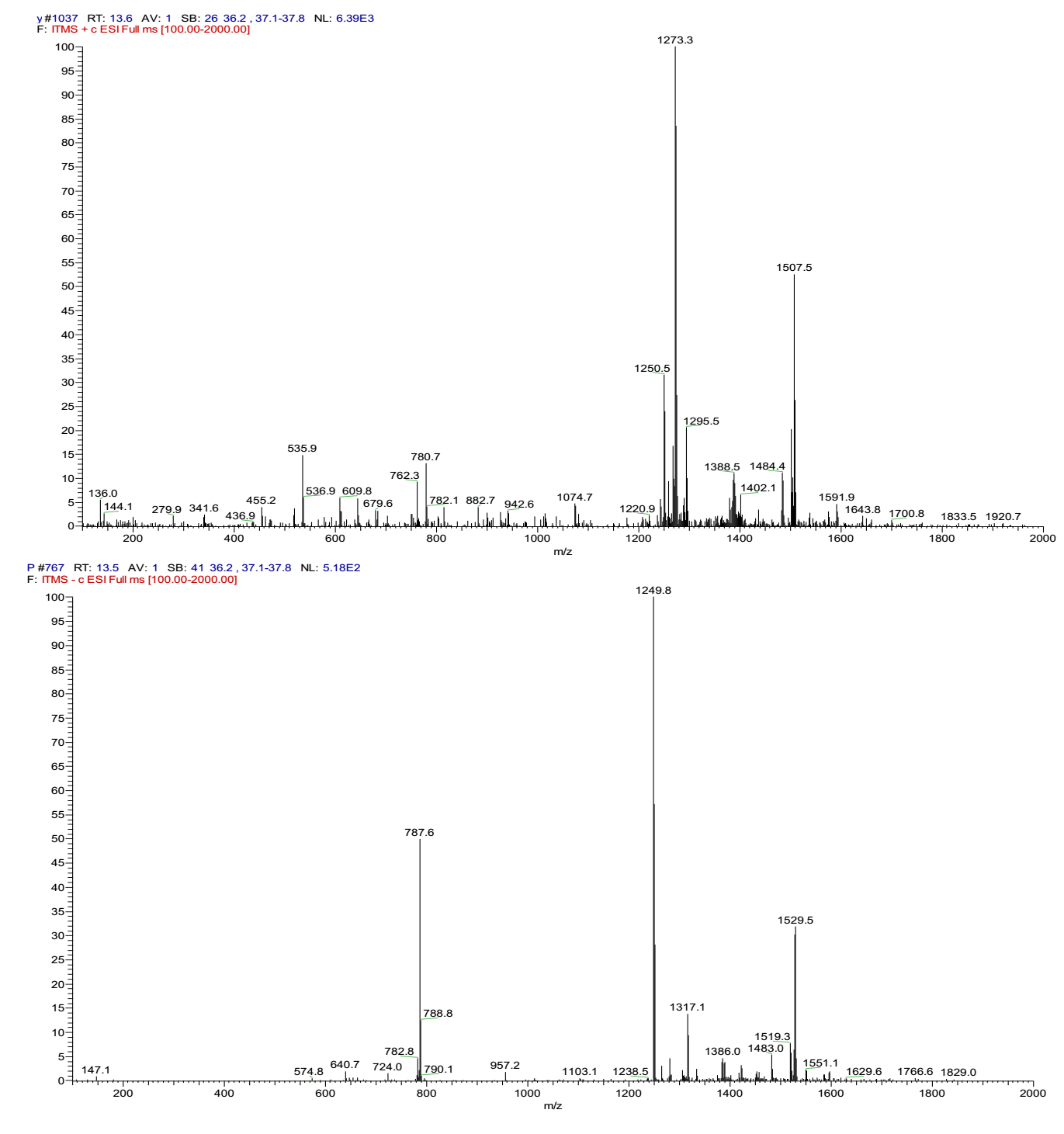


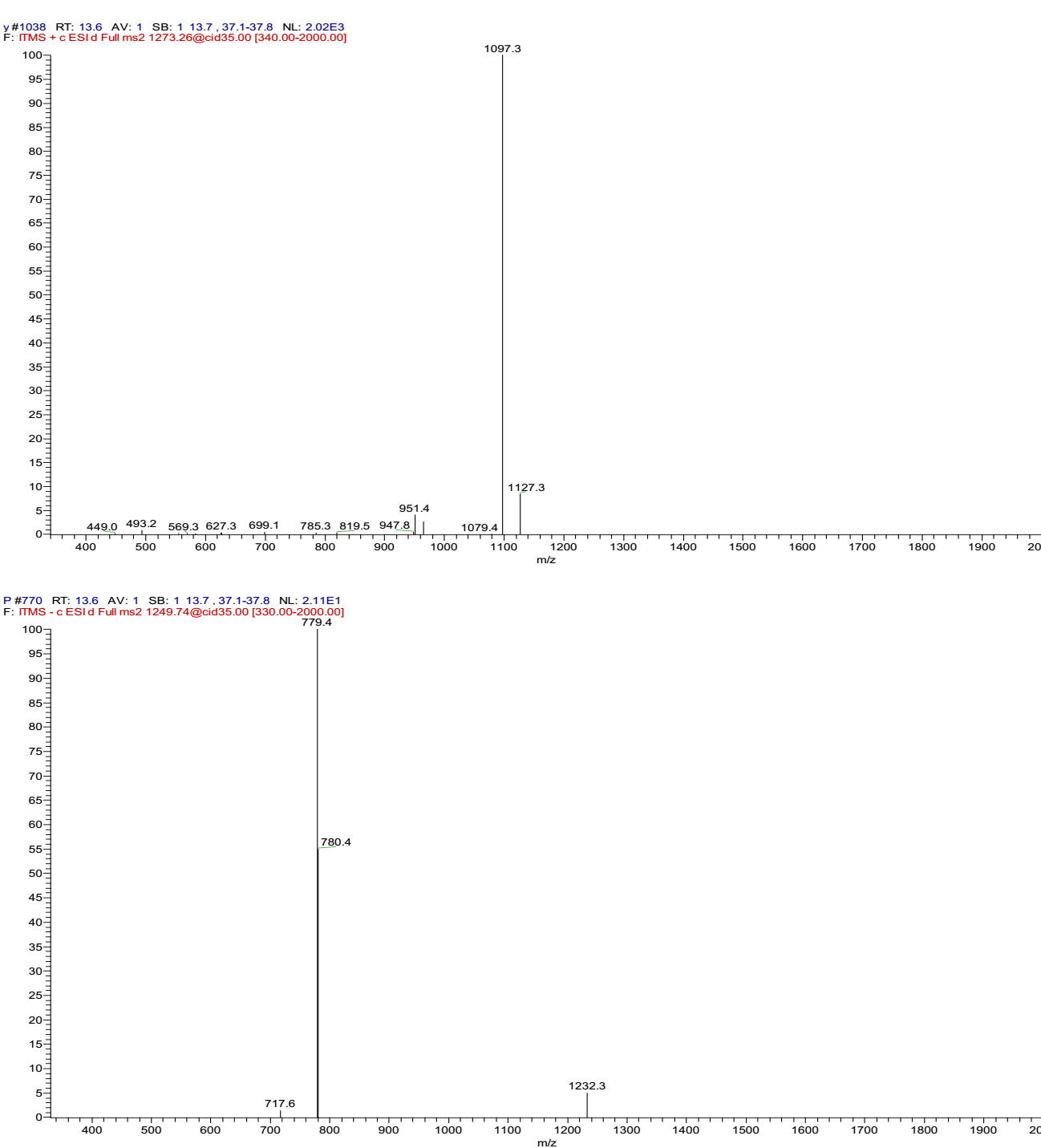


Figure 6: MS spectra of compound **6**


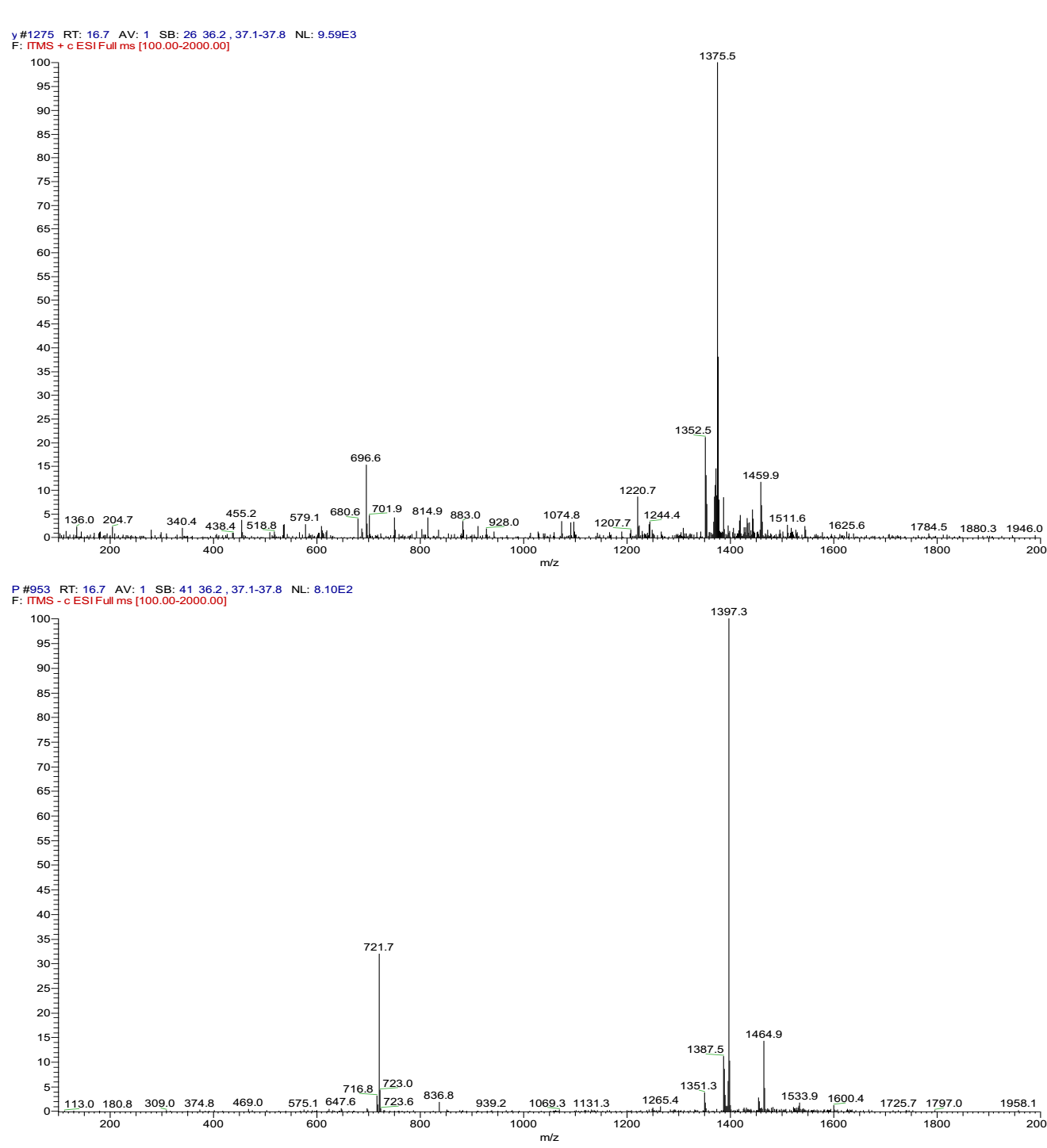


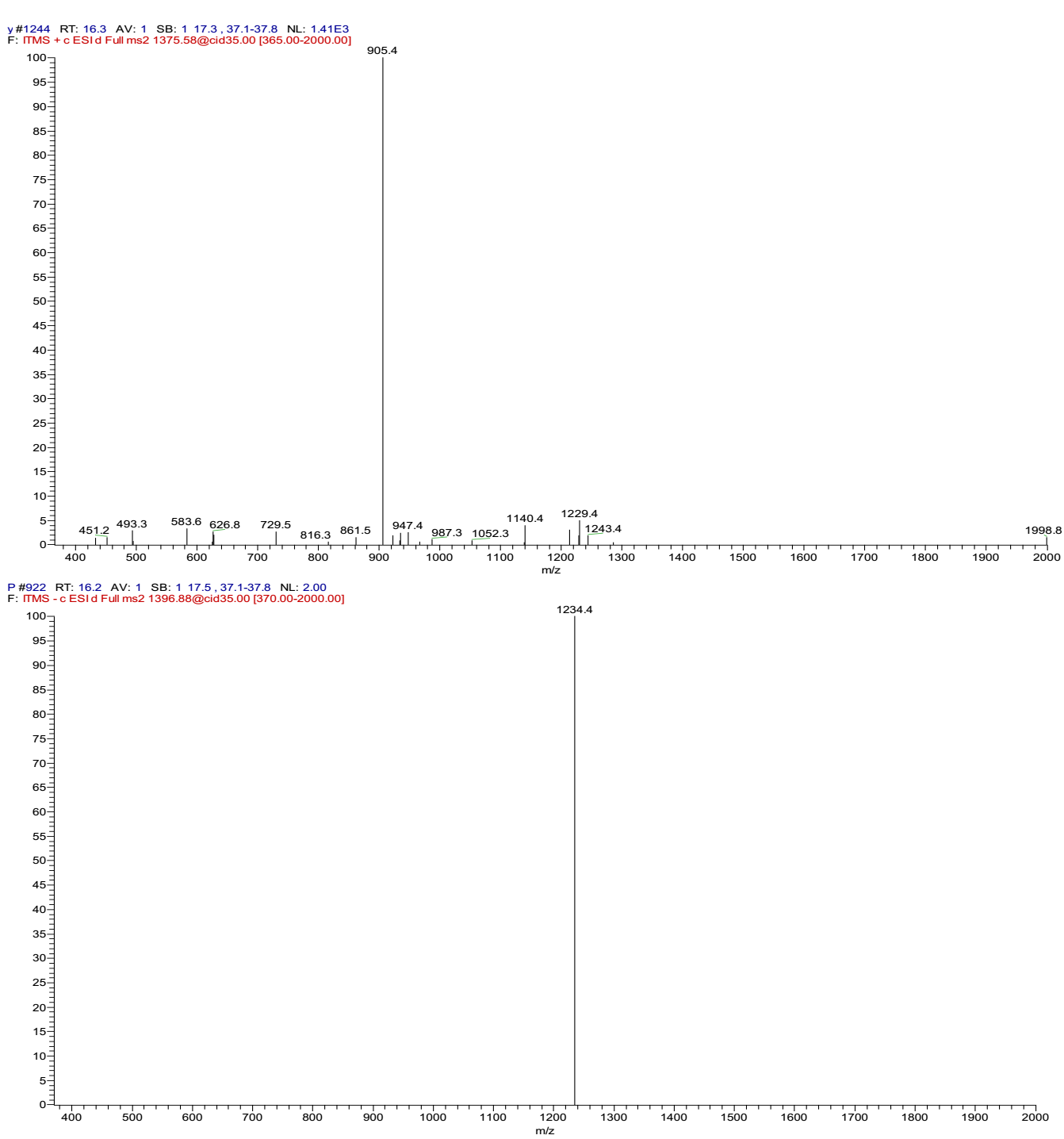


Figure 7: MS spectra of compound **7**


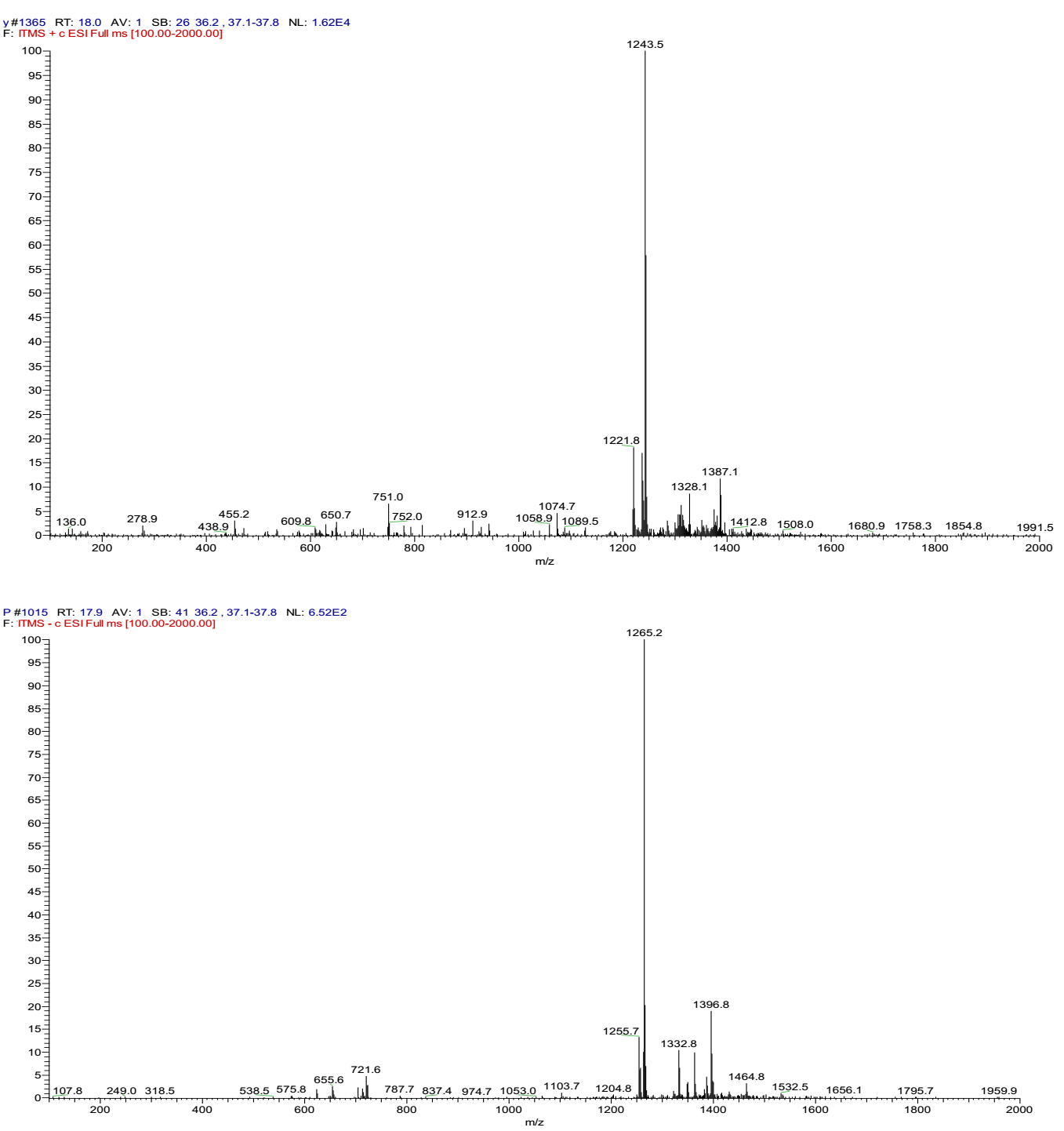


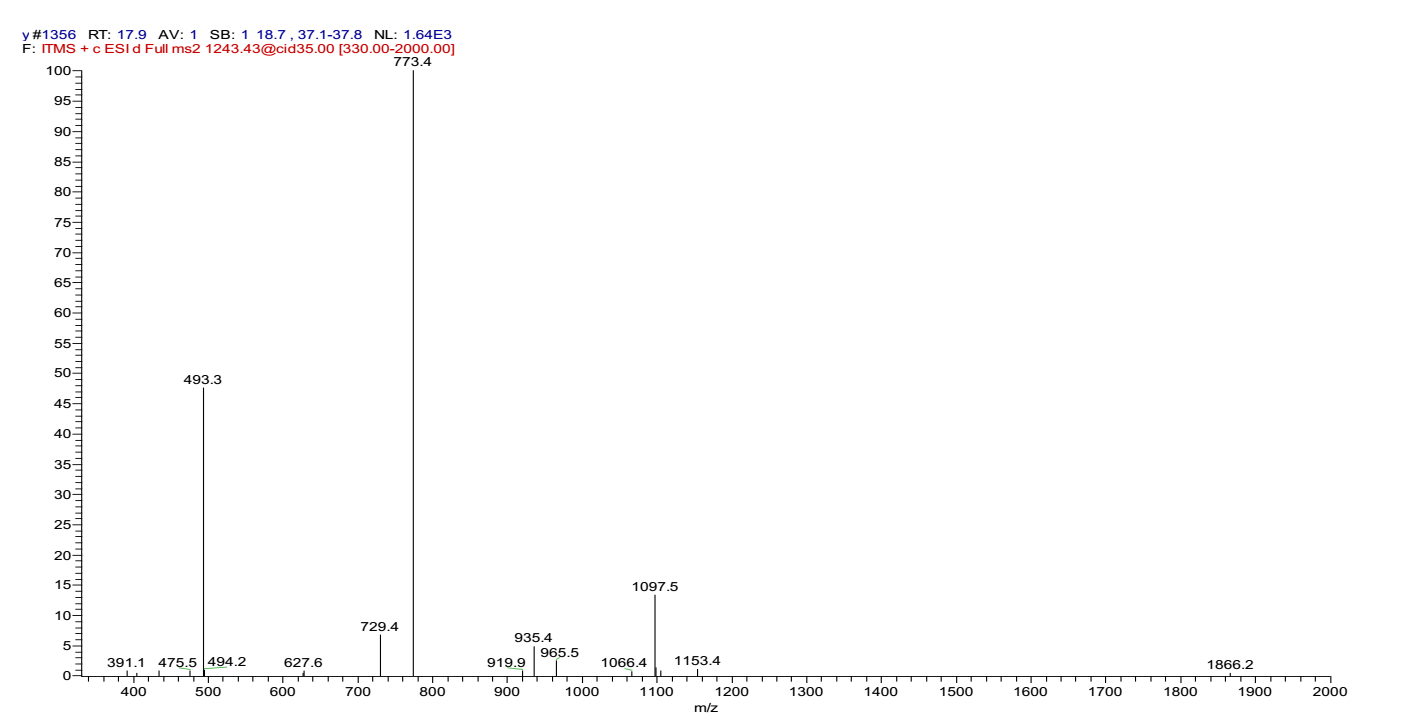


Figure 8: MS spectra of compound **8**


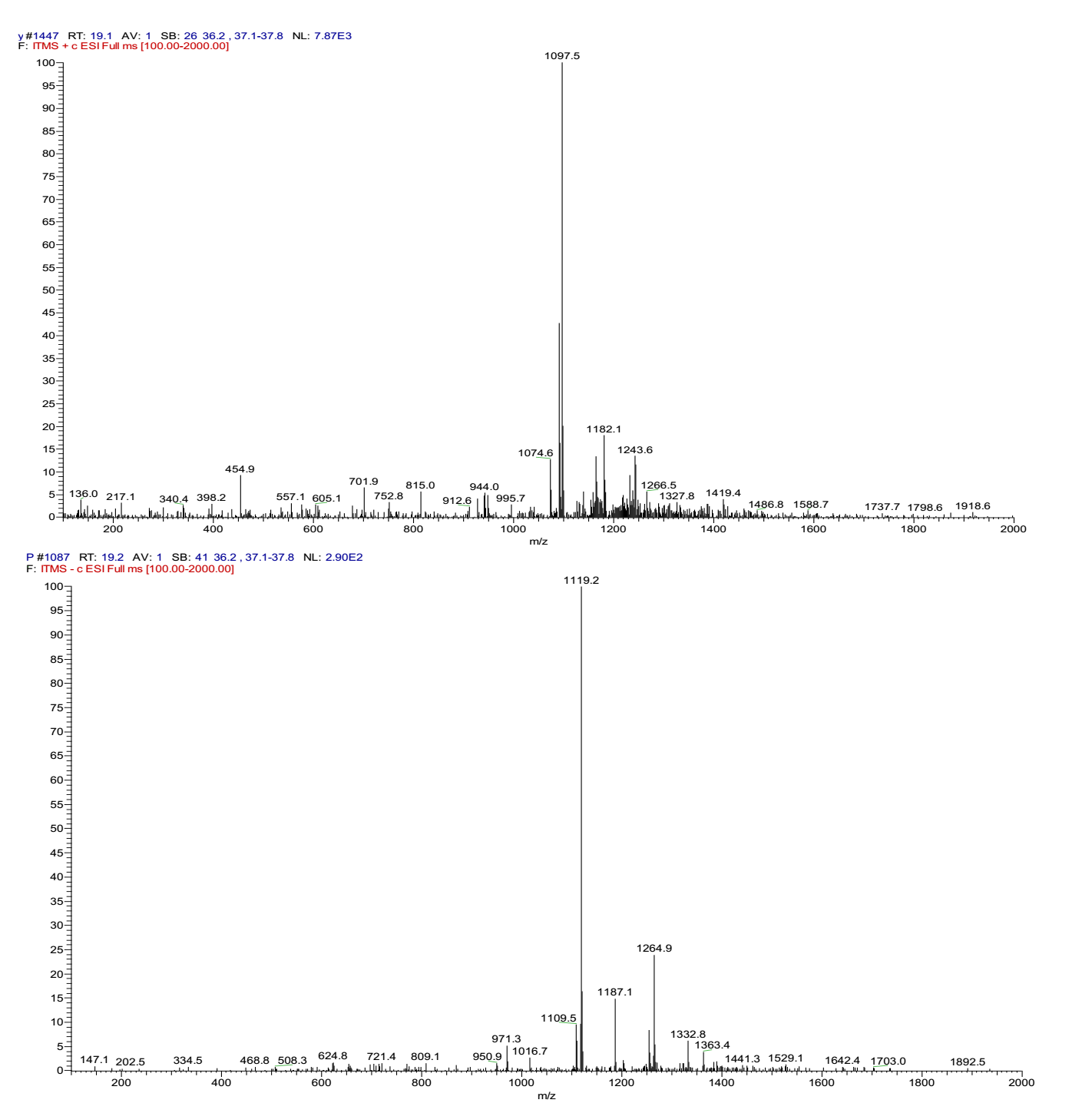


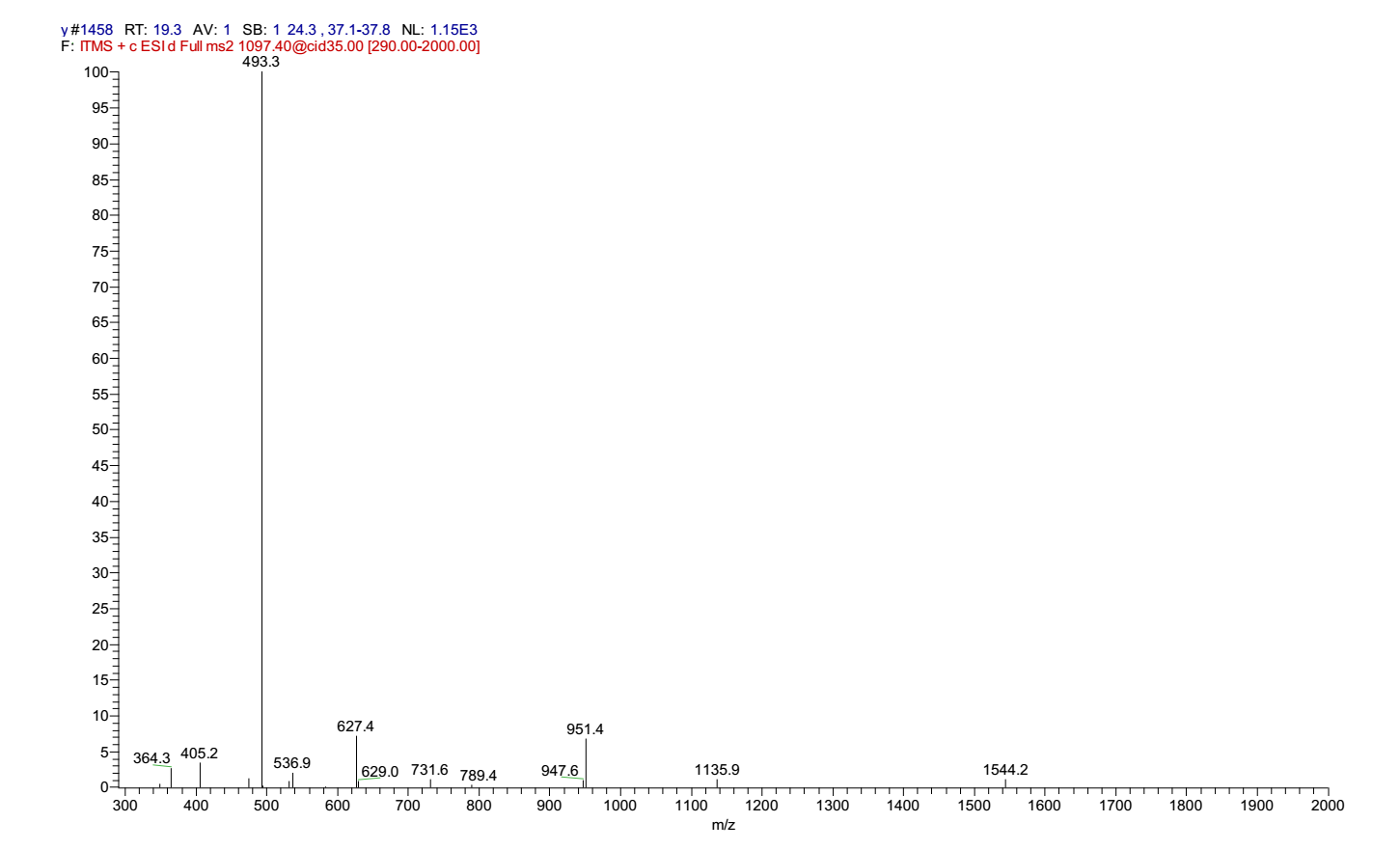


Figure 9: MS spectra of compound **9**


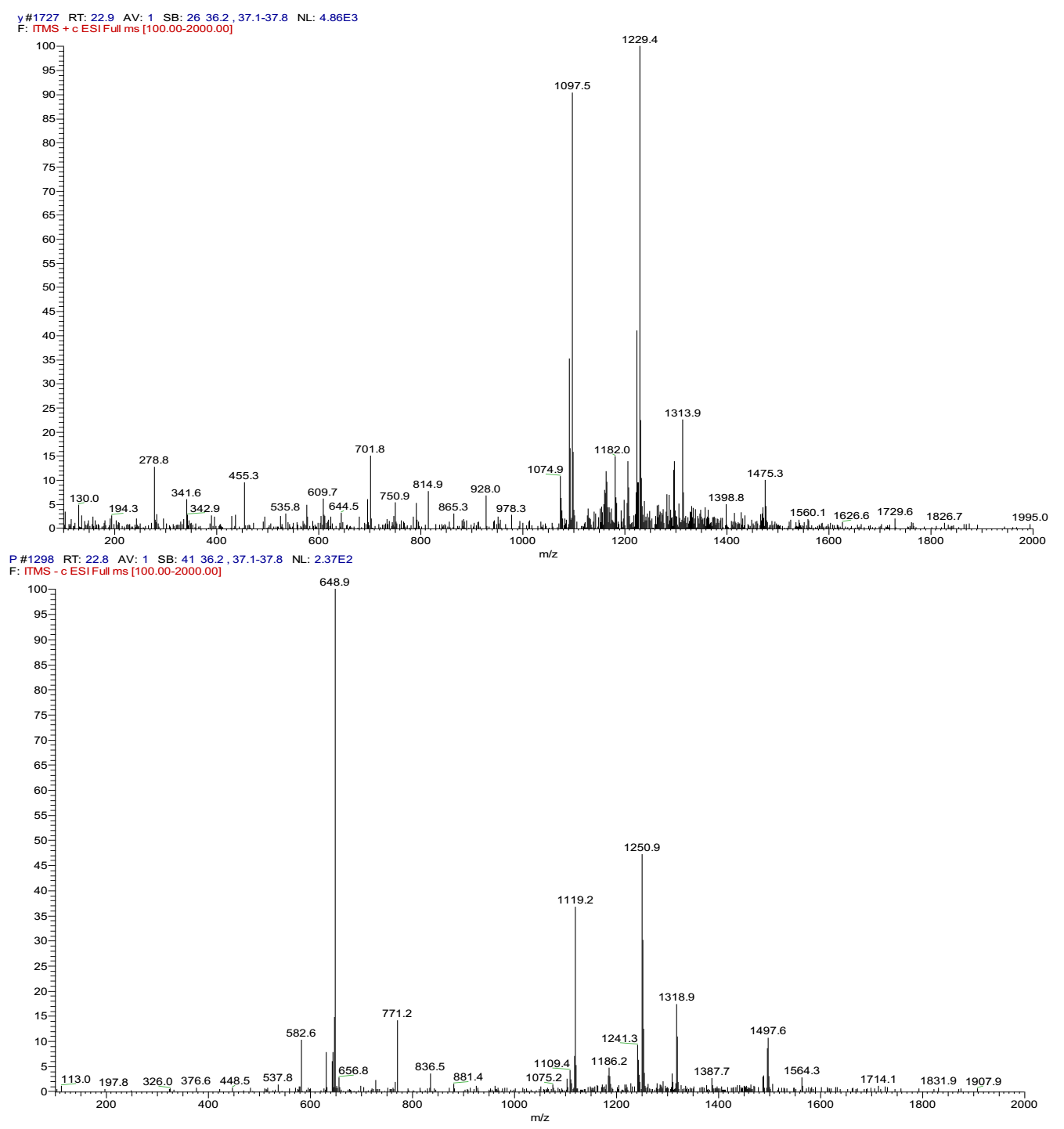


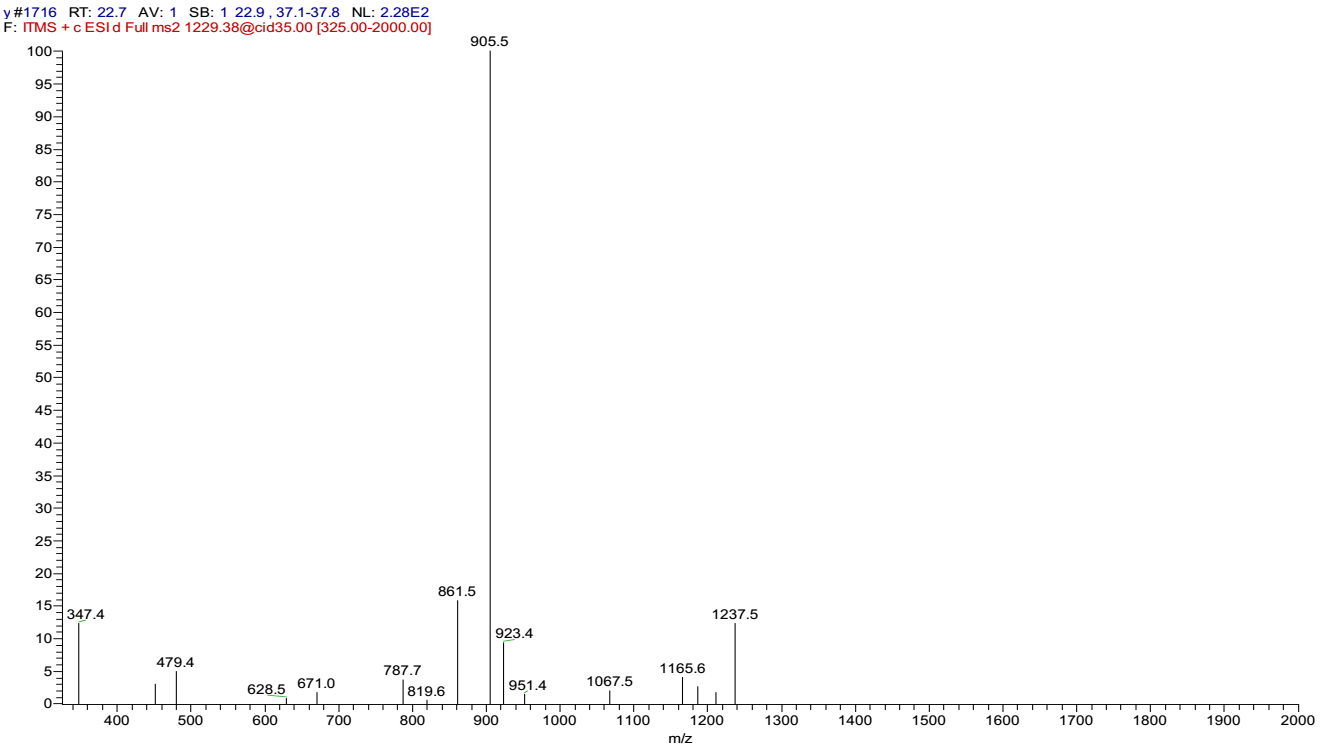


Figure 10: MS spectra of compound **10**


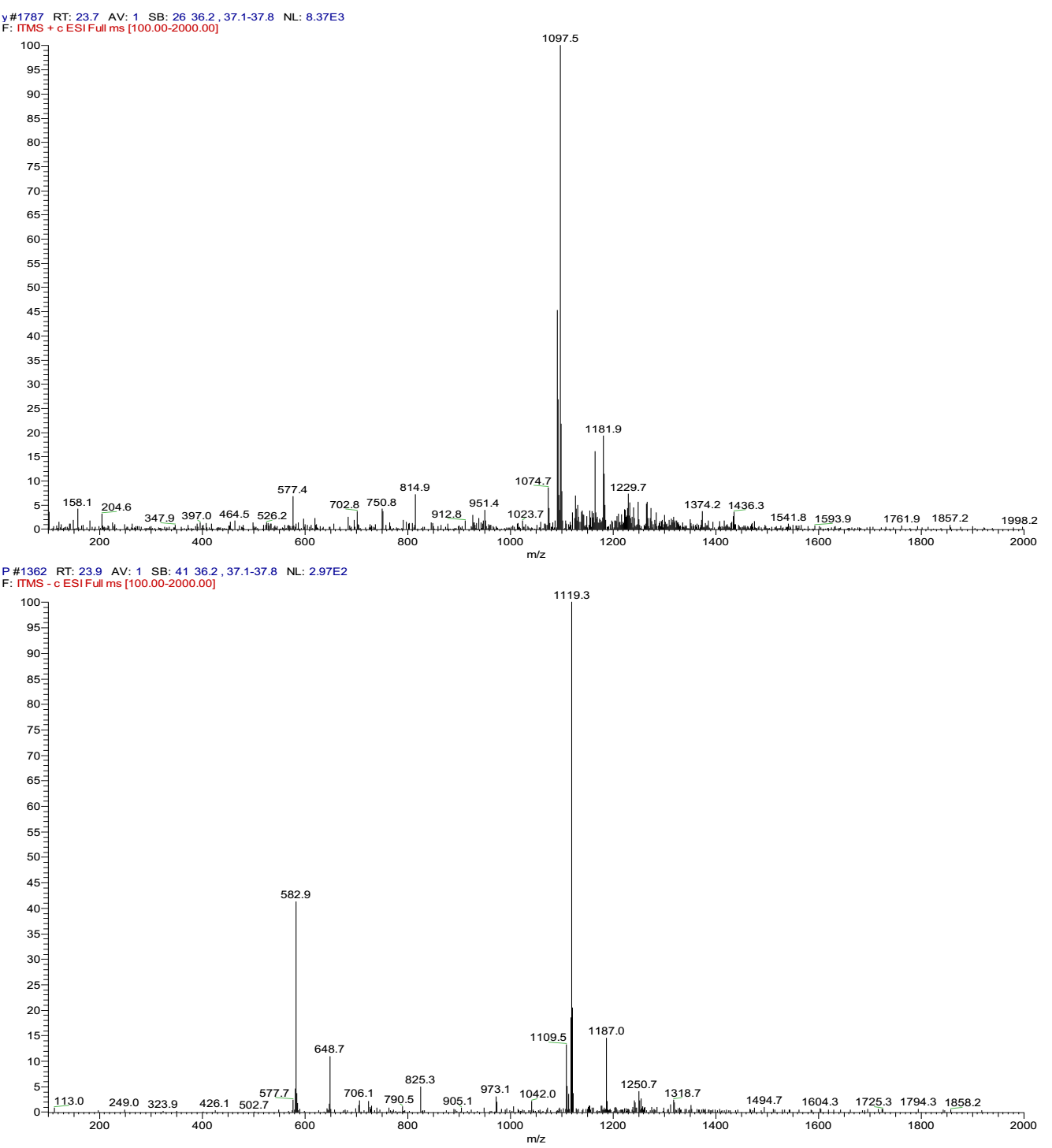


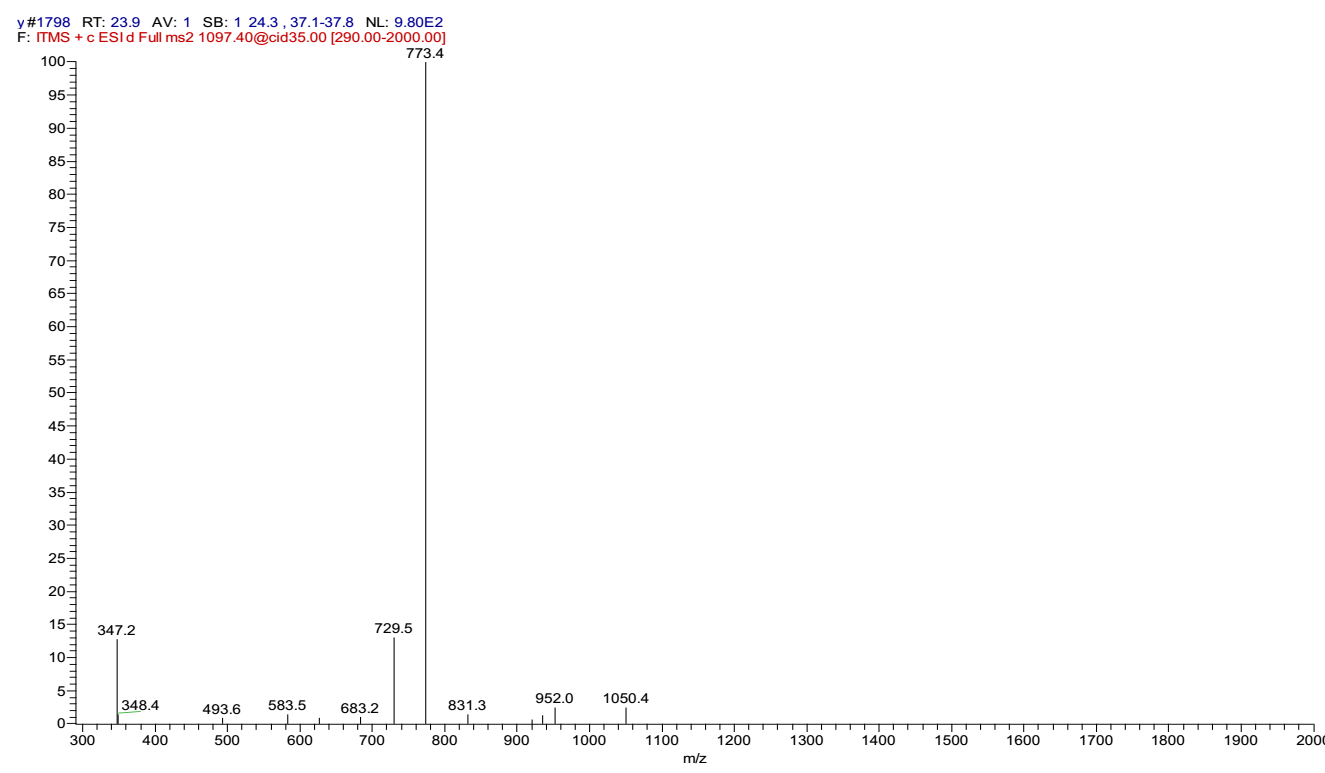


Figure 11: MS spectra of compound **11**


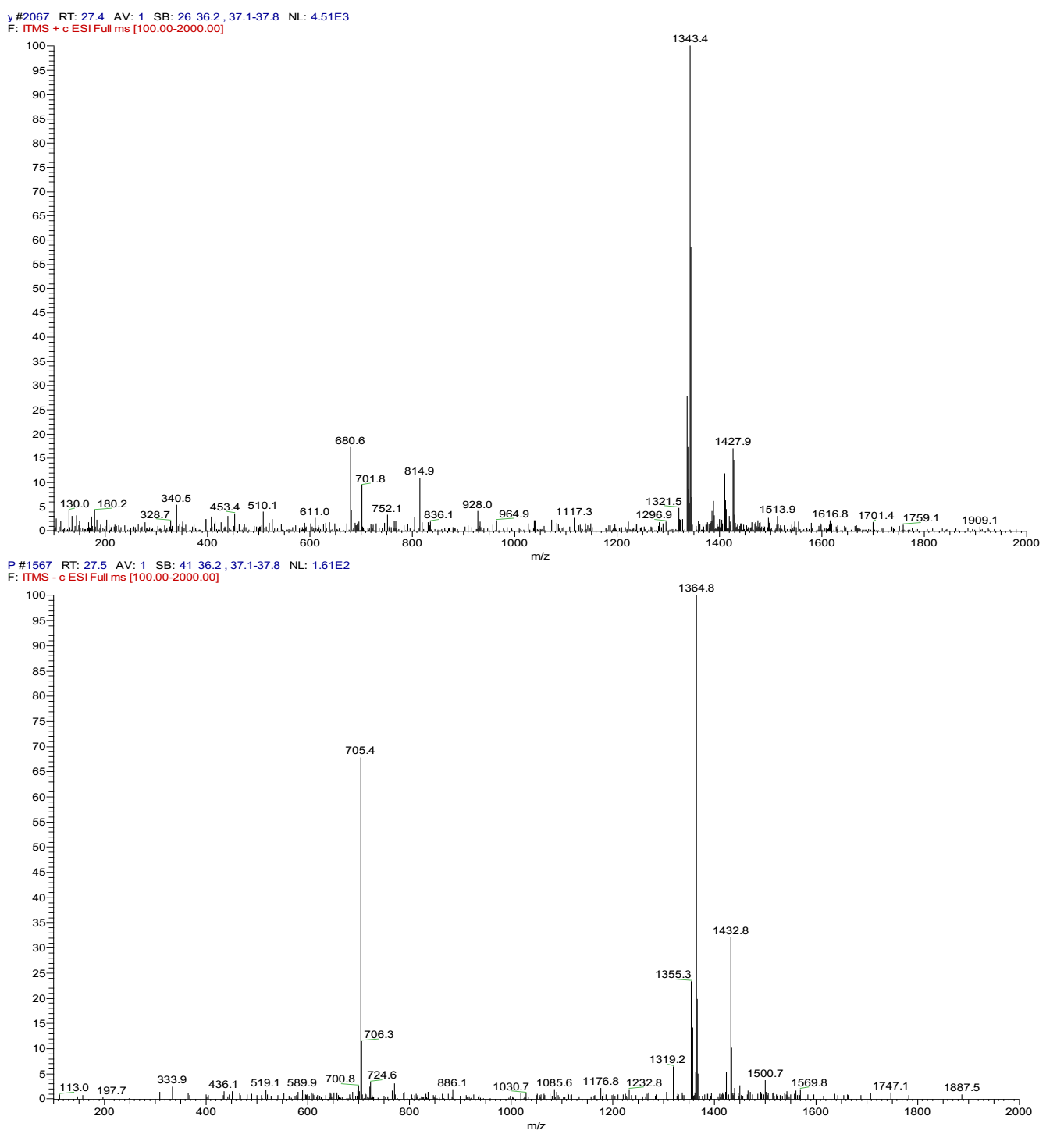


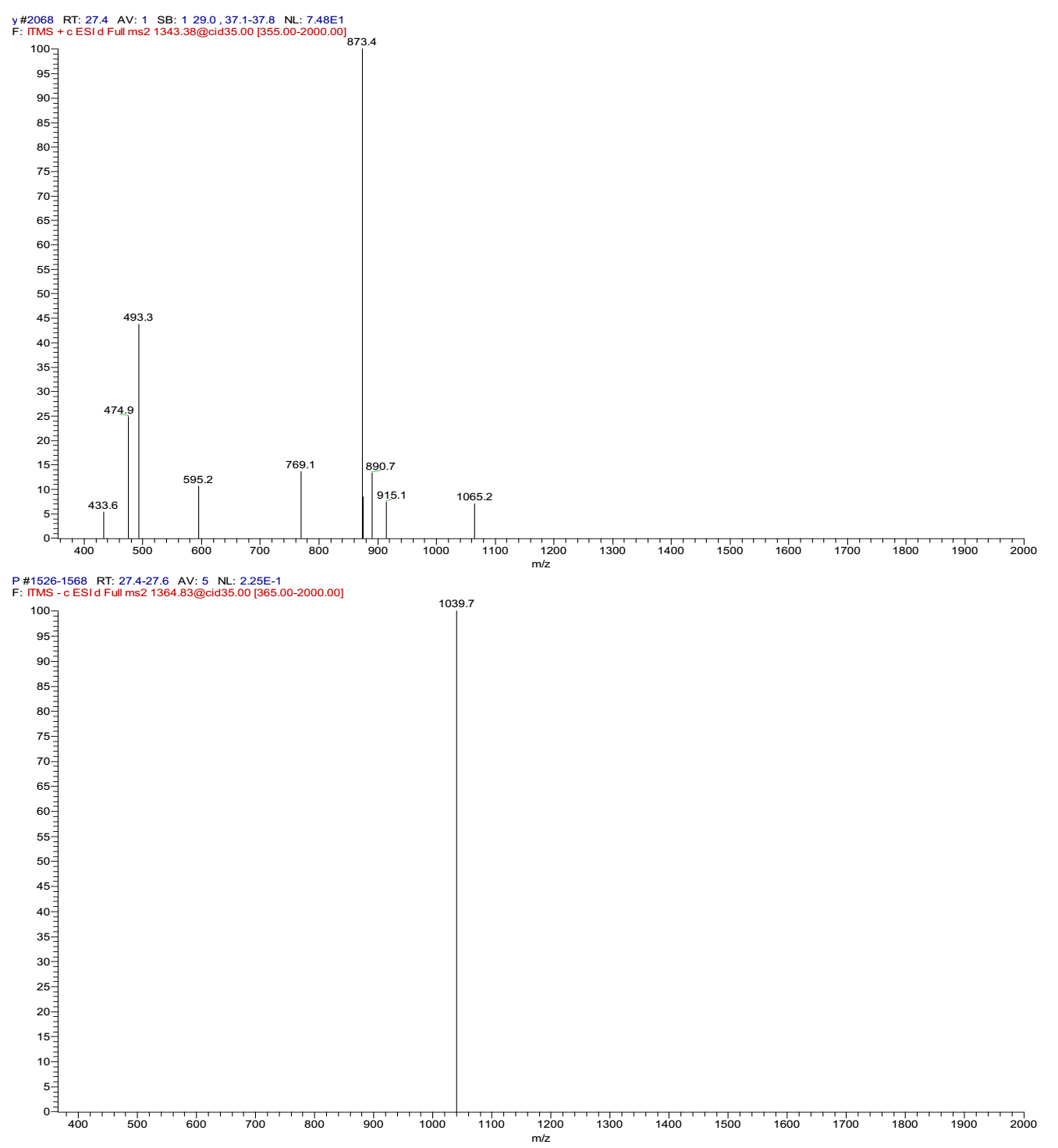


Figure 12: MS spectra of compound **12**


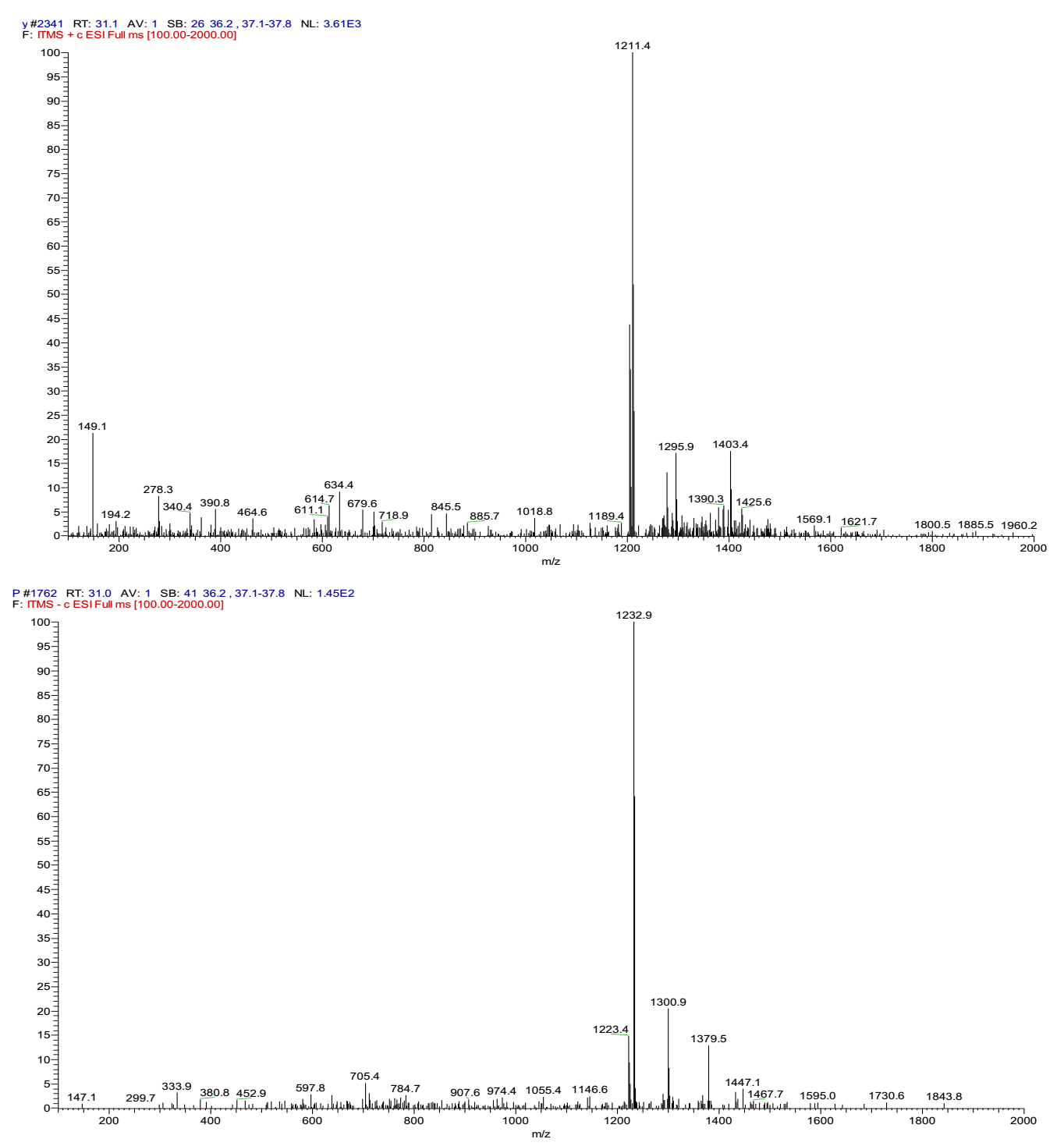


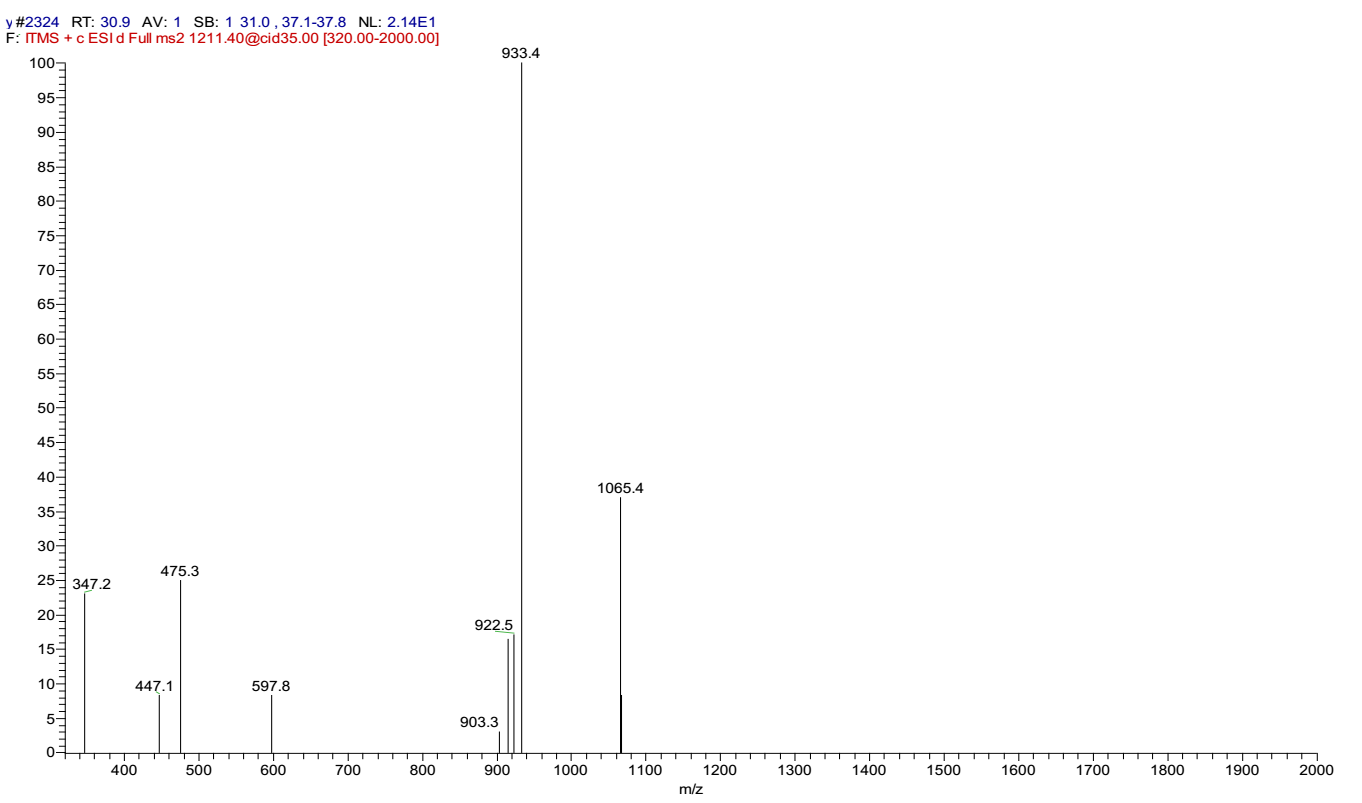


Figure 13: MS spectra of compound **13**


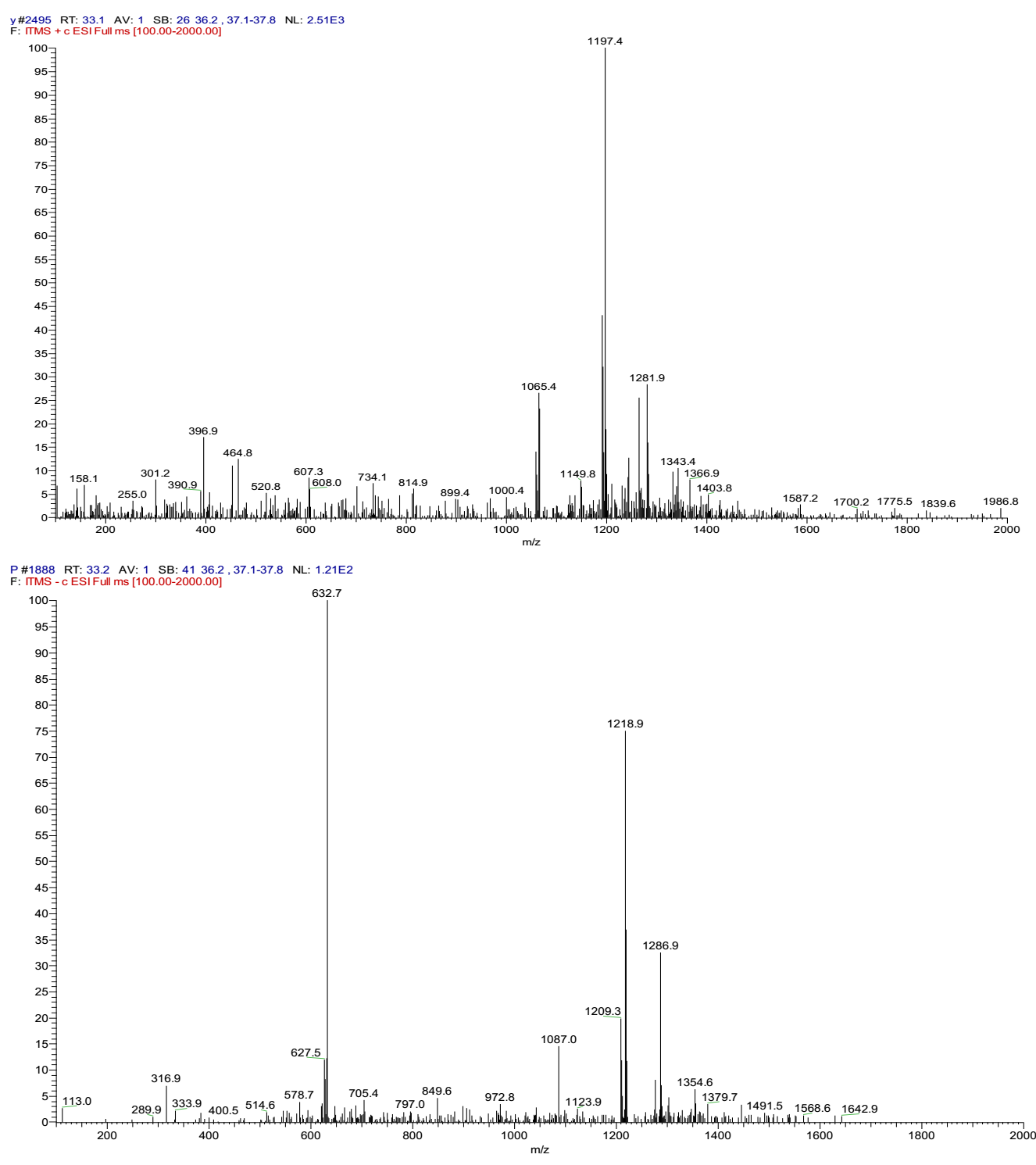


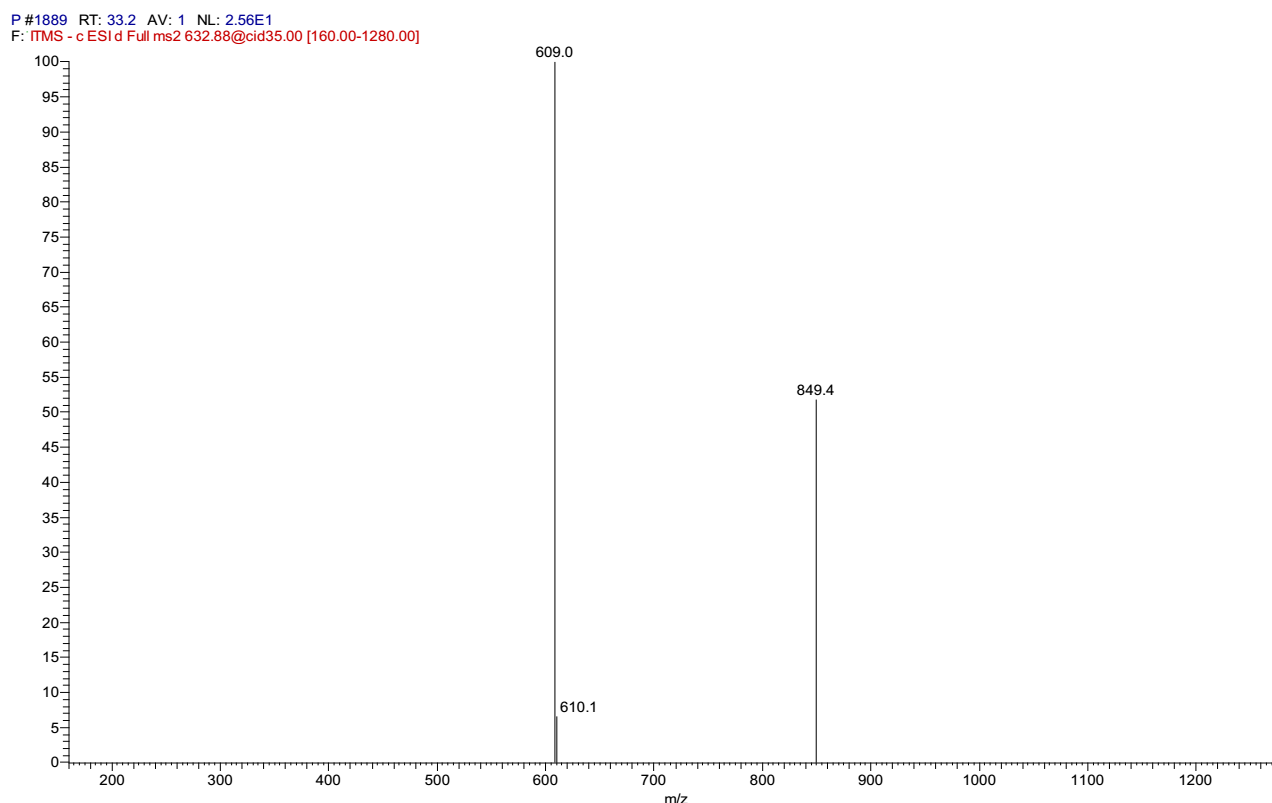


Figure 14: MS spectra of compound **14**


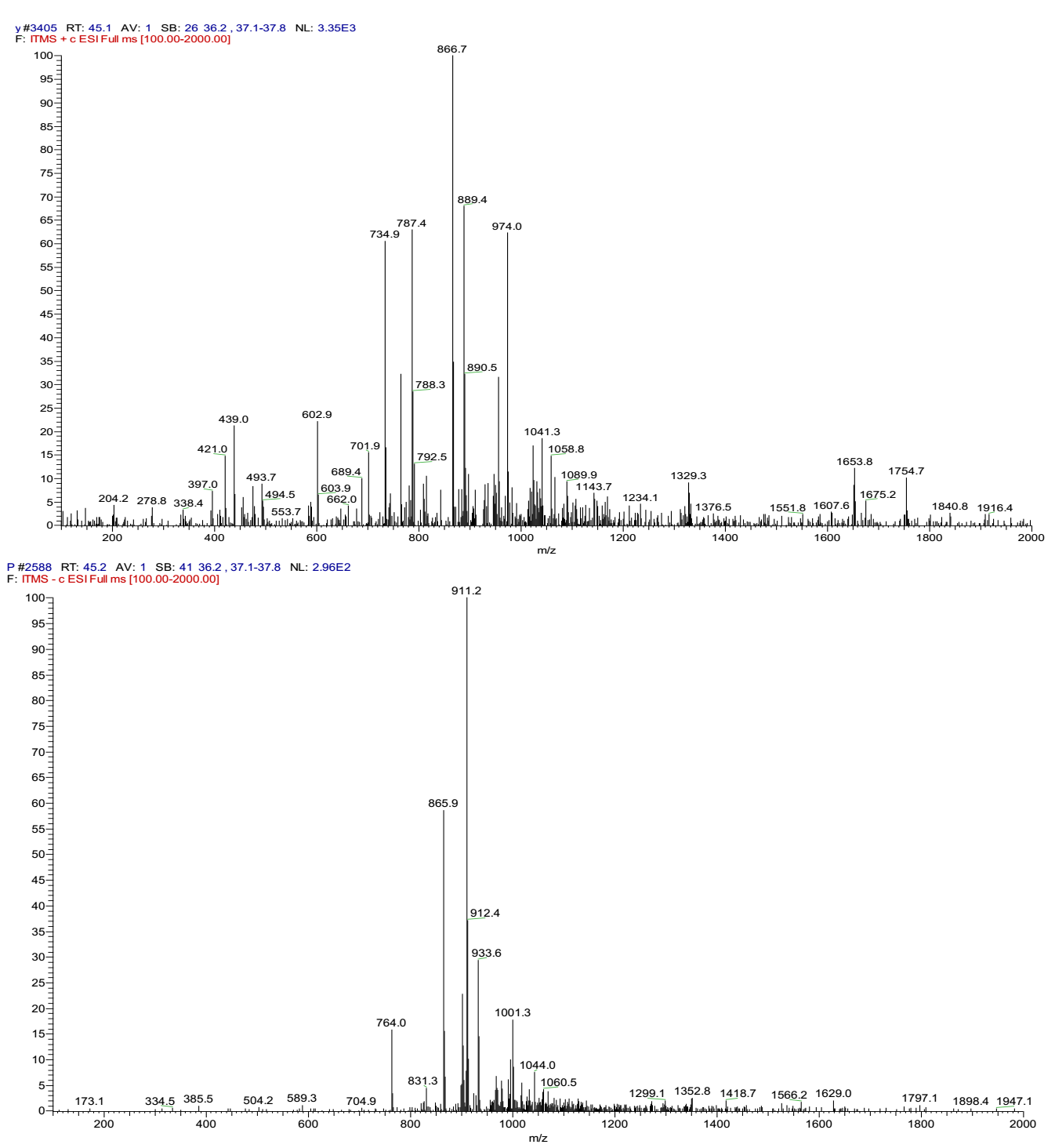


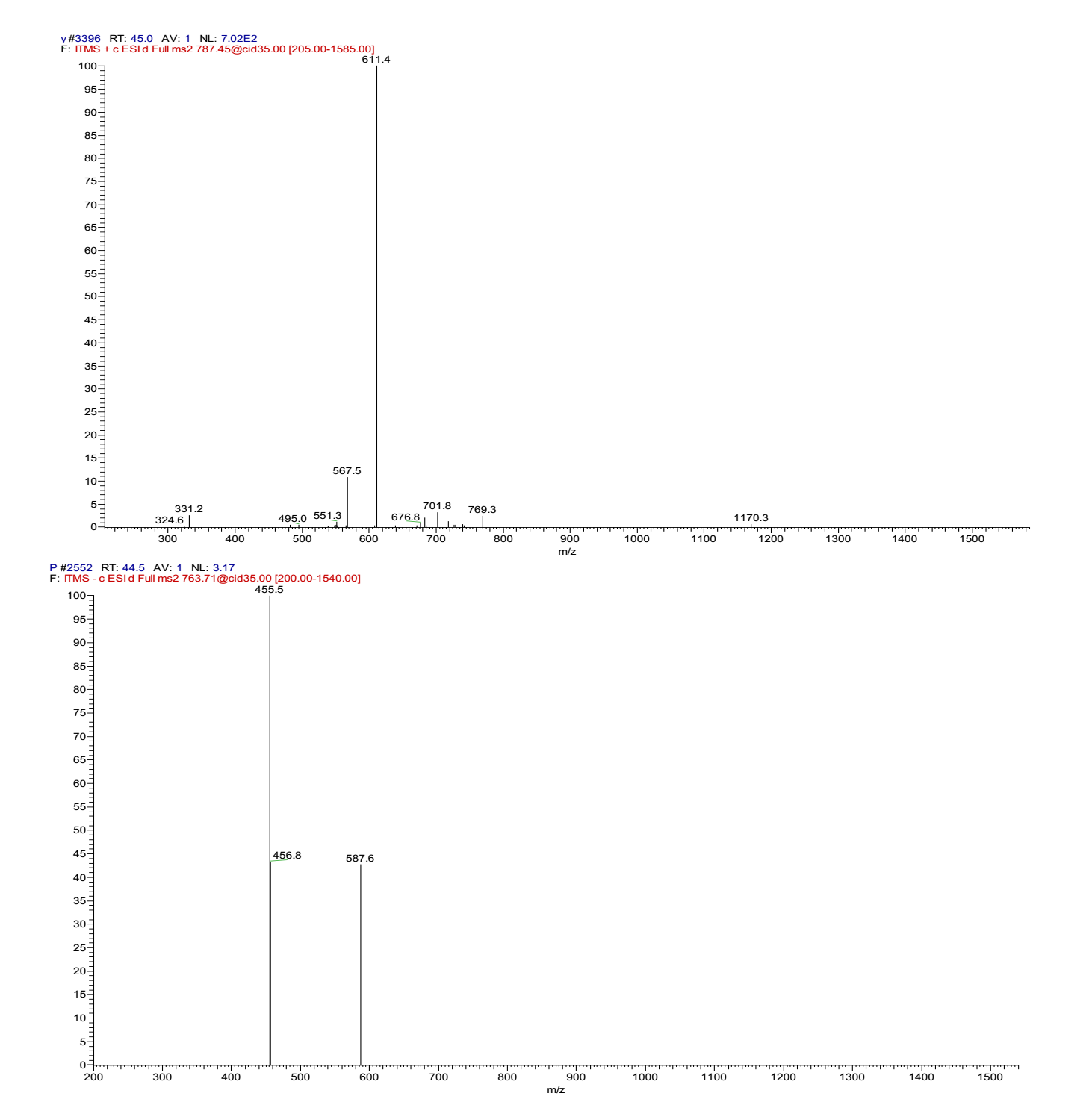


Figure 15: MS spectra of compound **15**


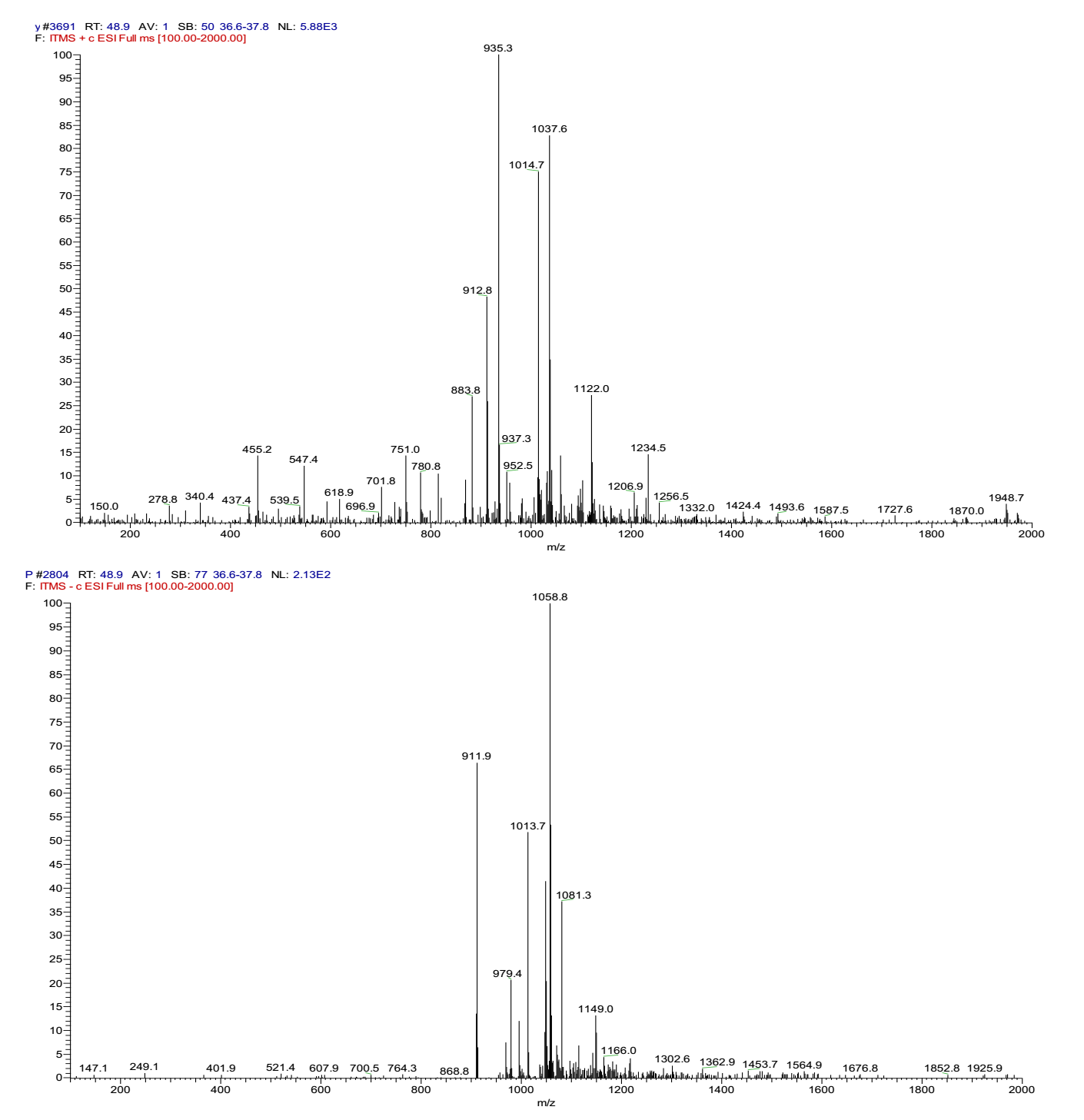


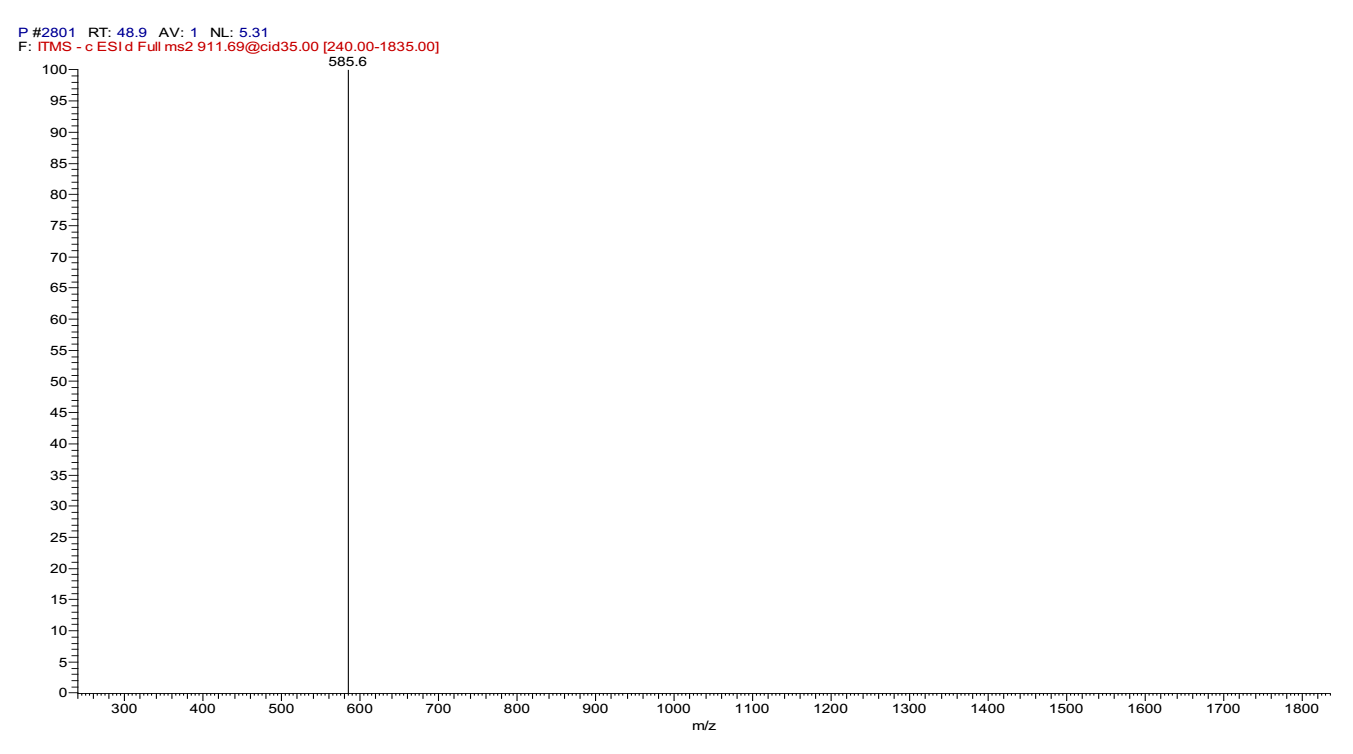


Figure 16: MS spectra of compound **16**


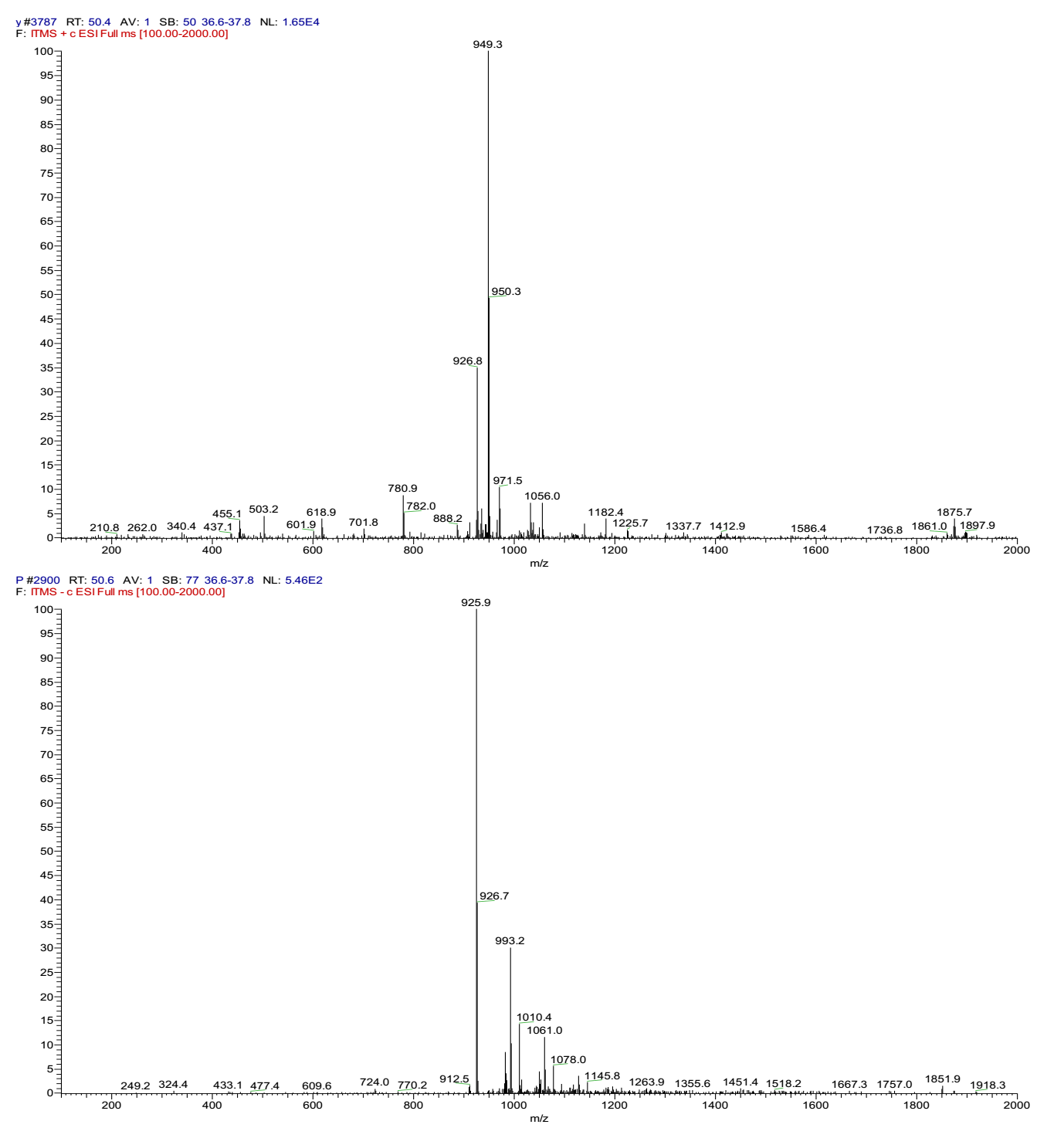


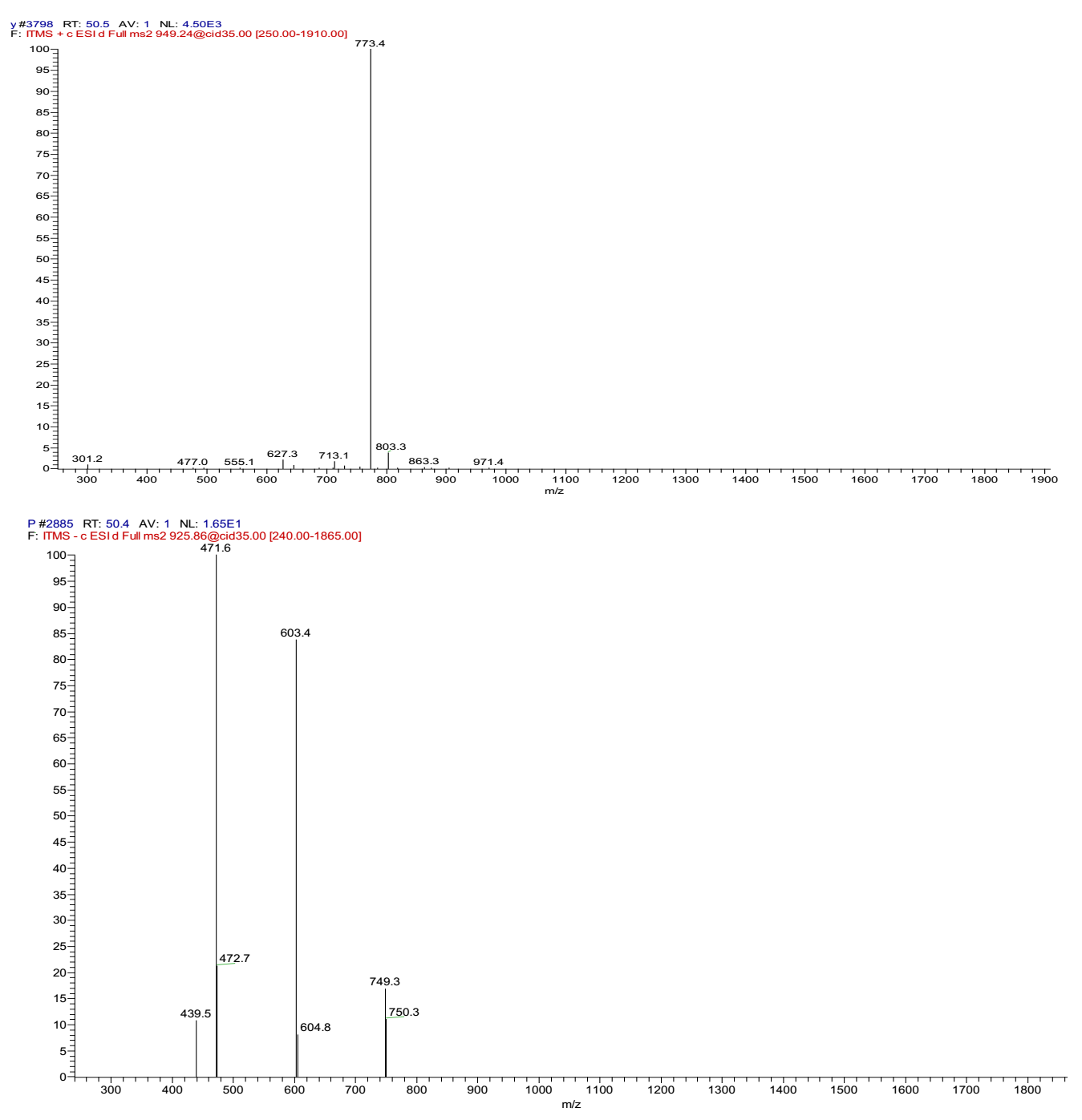


Figure 17: MS spectra of compound **17**


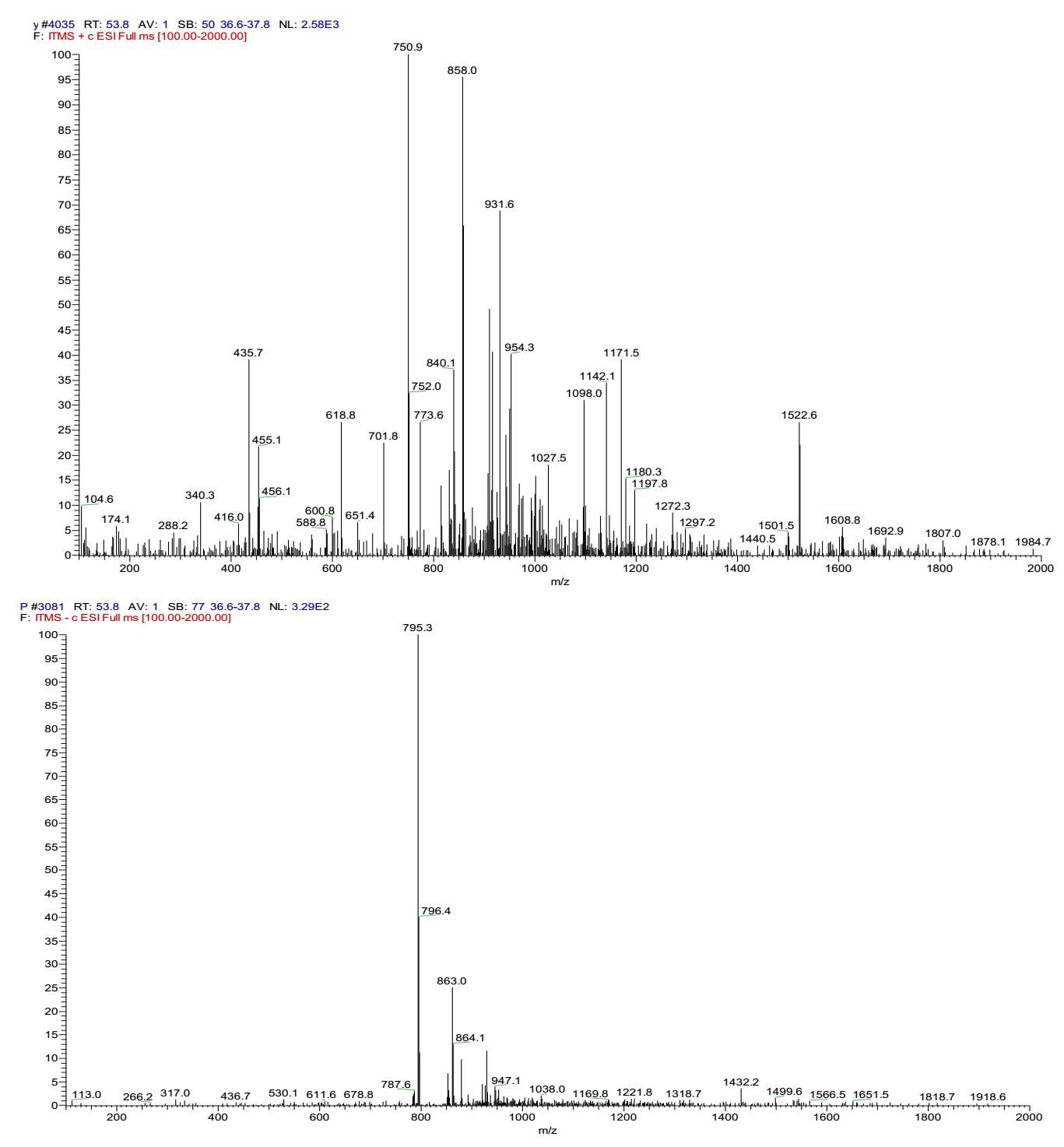


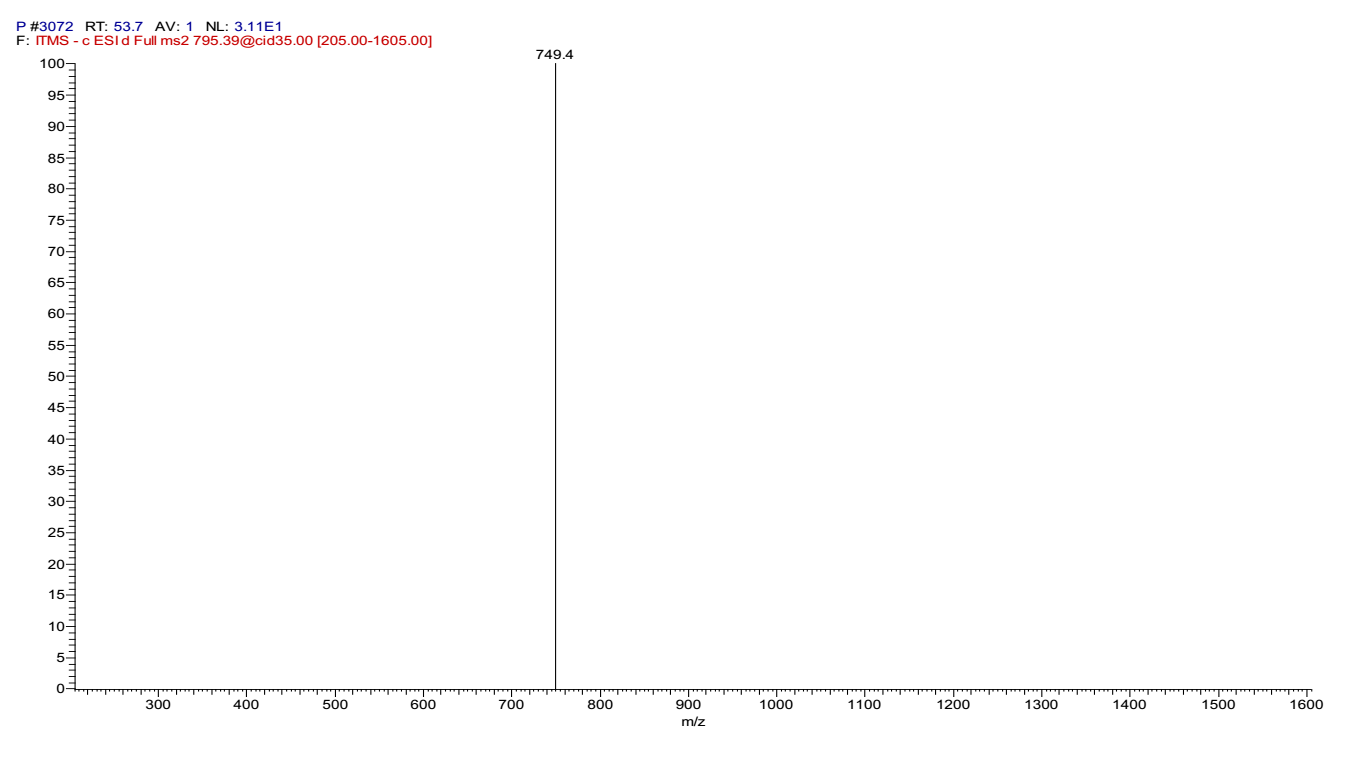


Figure 18: MS spectra of compound **18**


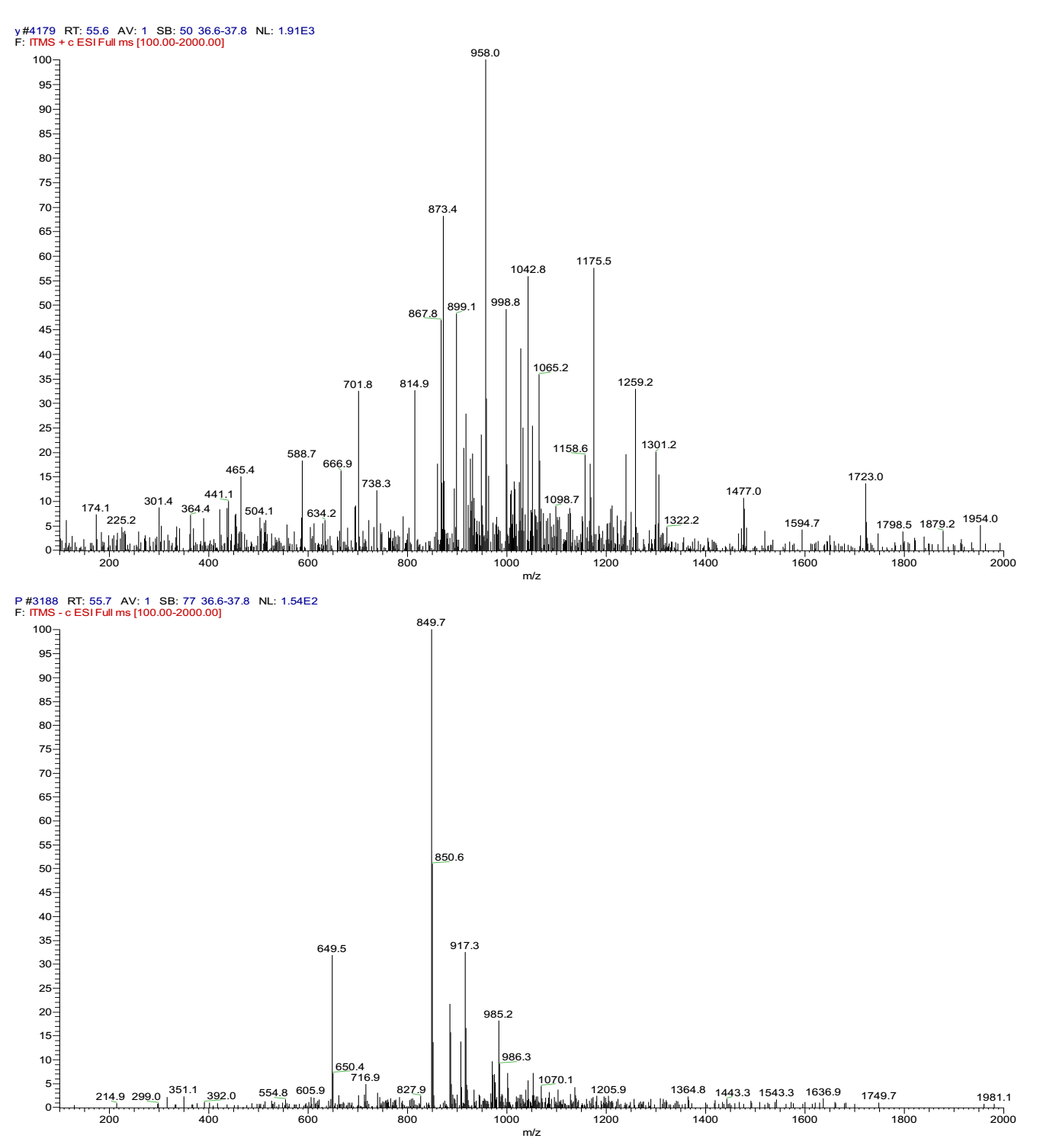


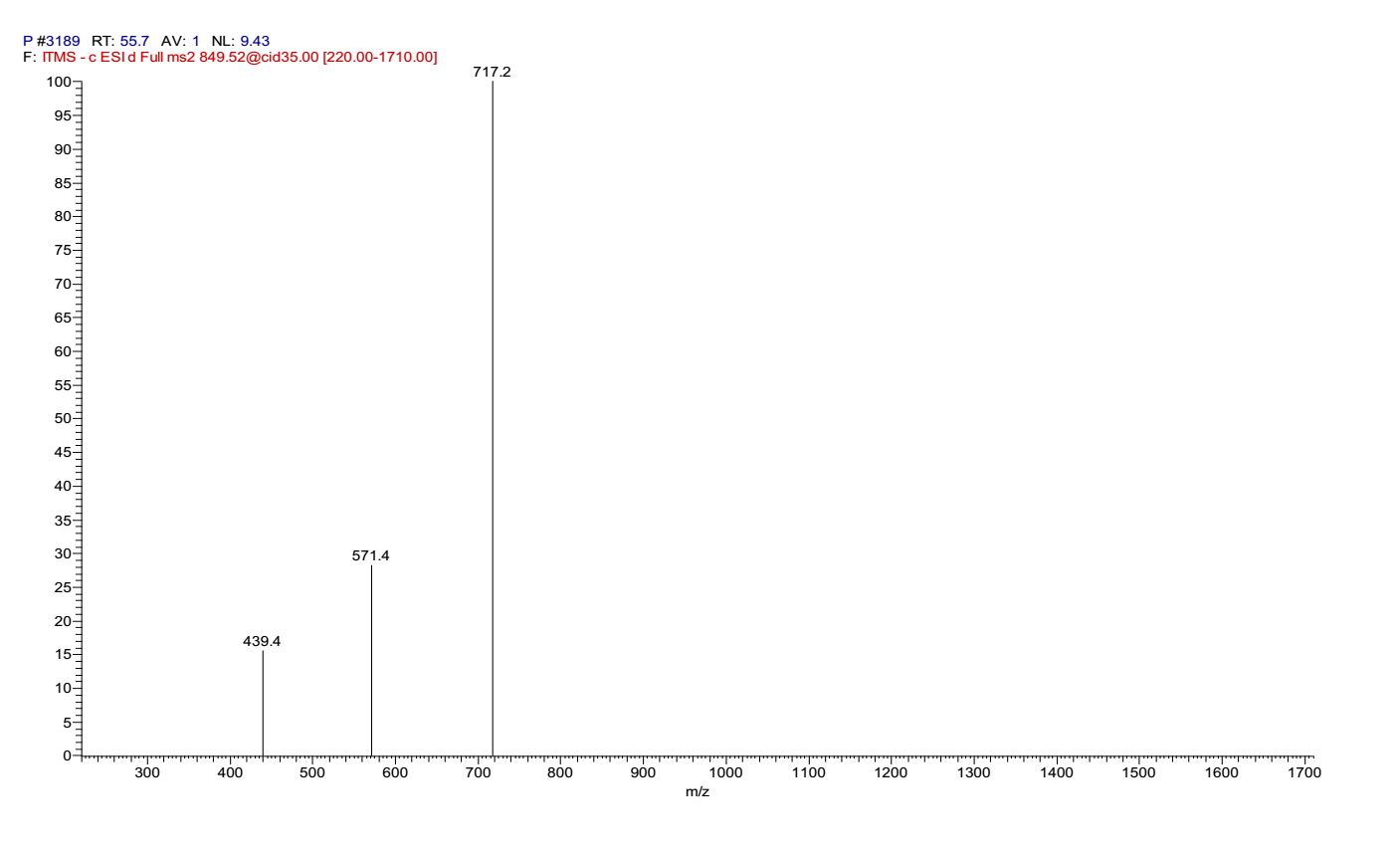


Figure 19: MS spectra of compound **19**


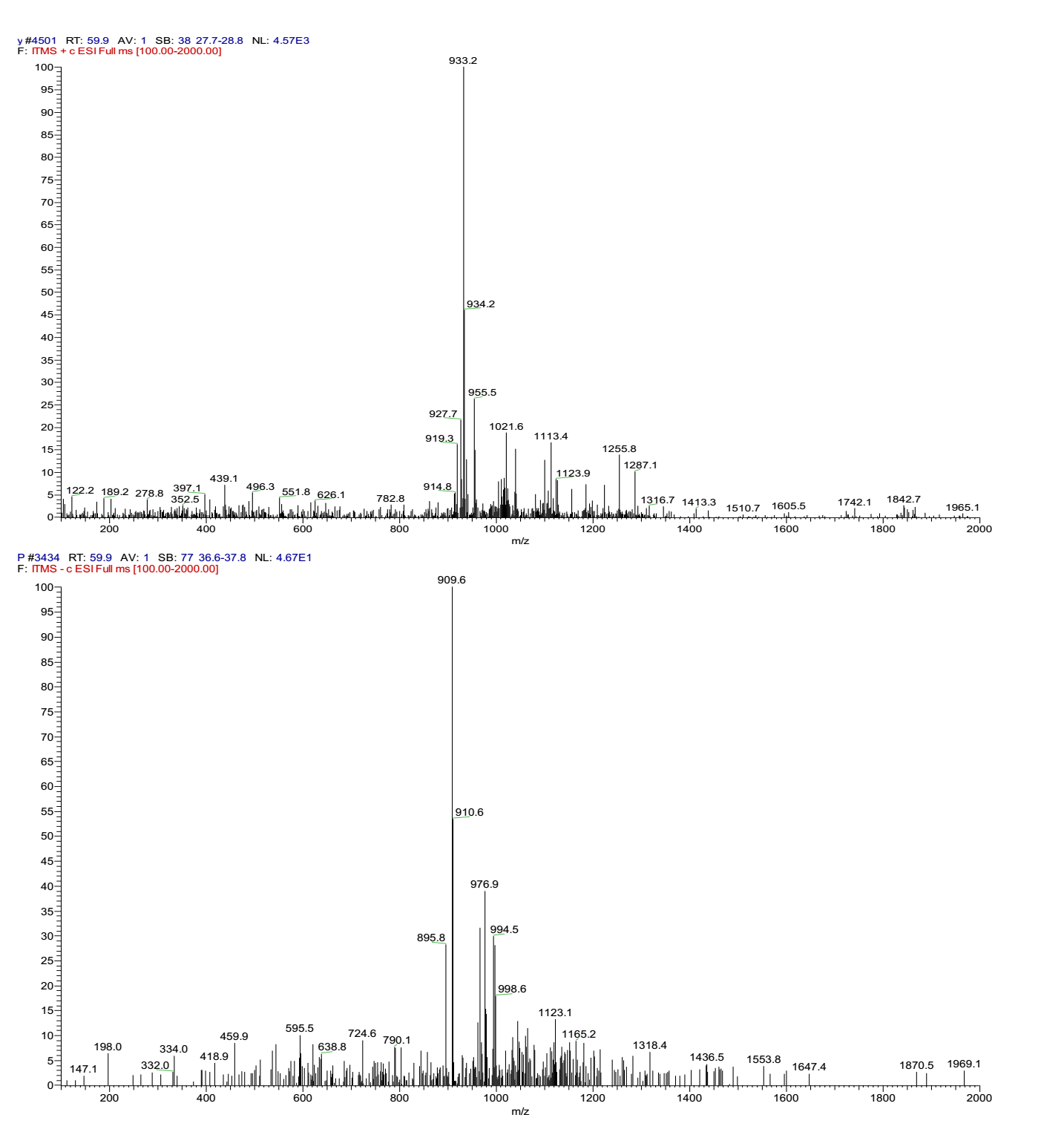


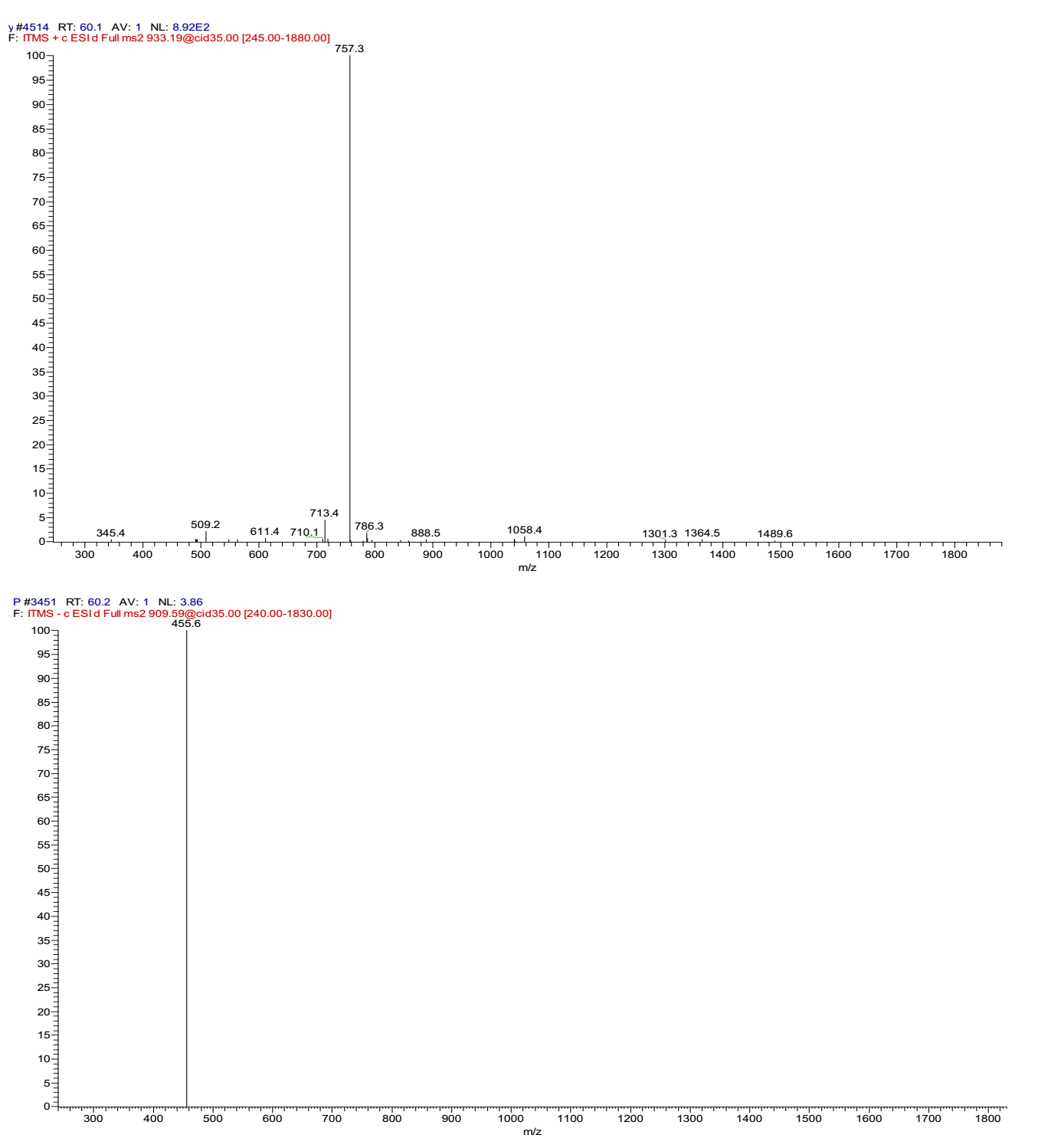


Figure 20: MS spectra of compound **20**


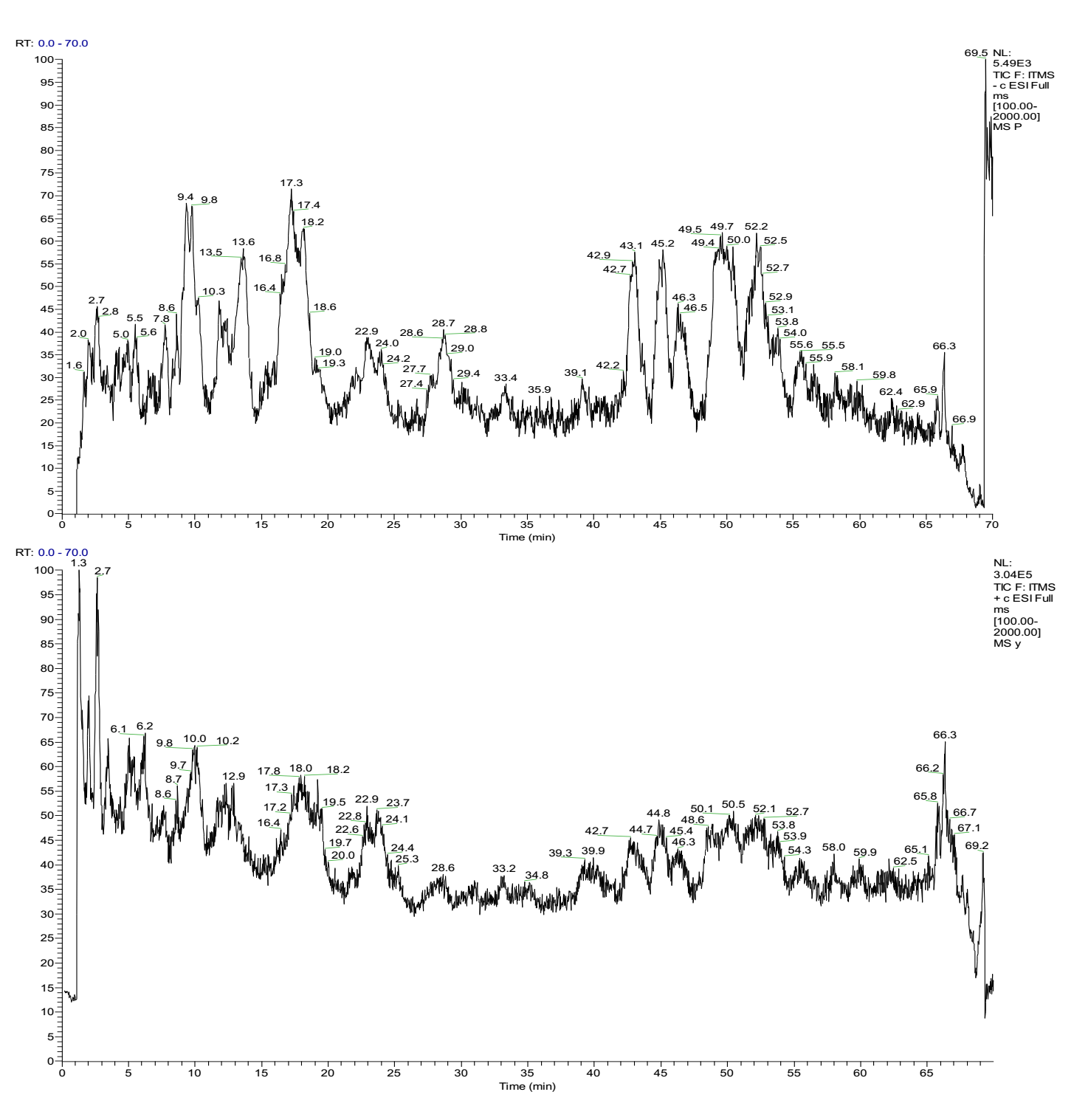


Figure 21: MS total ion current (TIC) chromatogram of TSS

**Table 1: Triterpenoid saponins identified in the TSS by HPLC-ESI-MS/MS**

| Peak No. | *t*_R_ (min) | Molecular formula | ESI-MS | ESI-MS^2^ | Identification |
| --- | --- | --- | --- | --- | --- |
| 1 | 7.7 | C_58_H_90_O_28_ | 1257 [M + Na]^+^ | 1081 [M + Na-176]^+^  935 [M + Na-176-146]^+^  611 [(M + Na-176)-470]^+^  493 [470+Na]^+^ | Stauntoside K |
| 2 | 9.2 | C_63_H_100_O_30_ | 1359 [M + Na]^+^ | 889 [M + Na-470]^+^  845 [(M + Na-470)-44]^+^  757 [(M + Na-470)-132]^+^  611 [(M + Na-470)-132-146]^+^  493 [470+Na]^+^ | Yemuoside YM_28_ |
| 3 | 9.8 | C_58_H_92_O_26_ | 1249 [M+HCOO]^-^  1227 [M + Na]^+^ | 1081 [M + Na-146]^+^  919 [M + Na-146-162]^+^  757 [M + Na-470]^+^  713 [(M + Na-470)-44]^+^  611 [(M + Na-470)-132-146]^+^  493 [470+Na]^+^ | Glycoside L-H3 |
| 4 | 10.2 | C_52_H_82_O_22_ | 1103 [M+HCOO]^-^  1081 [M + Na]^+^ | 935 [M + Na-146]^+^  611 [M + Na-470]^+^  493 [470+Na]^+^ | Glycoside L-G1 |
| 5 | 12.3 | C_52_H_82_O_22_ | 1103 [M+HCOO]^-^  1081 [M + Na]^+^ | 919 [M + Na-162]^+^  757 [M + Na-162-162]^+^  713 [M+Na-162-162-44]^+^  347 [324+Na]^+^ | new |
| 6 | 13.6 | C_59_H_94_O_28_ | 1249 [M - H]^-^  1273 [M + Na]^+^ | 1097 [M + Na-176]^+^  951 [M + Na-176-146]^+^  819 [M + Na-176-146-132]^+^  627 [(M + Na-176)-470]^+^  493 [470+Na]^+^  779 [M-H-470]^-^ | Stauntoside L |
| 7 | 16.3 | C_64_H_104_O_30_ | 1397 [M+HCOO]^-^  1375 [M + Na]^+^ | 905 [M + Na-470]^+^  861 [(M + Na-470)-44]^+^  729 [(M + Na-470-44)-132]^+^  627 [(M + Na-470)-132-146]^+^  583 [(M + Na-470-44)-132-146]^+^  493 [470+Na]^+^ | Yemuoside YM_32_ |
| 8 | 17.9 | C_59_H_96_O_26_ | 1265 [M+HCOO]^-^  1243 [M + Na]^+^ | 1097 [M + Na-146]^+^  773 [M + Na-470]^+^  729 [(M + Na-470)-44]^+^  493 [470+Na]^+^ | Hederasaponin C |
| 9 | 19.3 | C_53_H_86_O_22_ | 1119 [M+HCOO]^-^  1097 [M + Na]^+^ | 951 [M + Na-146]^+^  627 [M + Na-470]^+^  493 [470+Na]^+^ | Hederasaponin D |
| 10 | 22.7 | C_58_H_94_O_26_ | 1251 [M+HCOO]^-^  1229 [M + Na]^+^ | 905 [M + Na-324]^+^  861 [(M + Na-324)-44]^+^  787 [(M + Na-132-146-132-18]^+^  347 [324 + Na]^+^ | Yemuoside YM_35_ |
| 11 | 23.9 | C_53_H_86_O_22_ | 1119 [M+HCOO]^-^  1097 [M + Na]^+^ | 773 [M + Na-470]^+^  729 [(M + Na-470)-44]^+^  347 [324 + Na]^+^ | Dipsacoside B |
| 12 | 27.4 | C_63_H_100_O_29_ | 1365 [M+HCOO]^-^  1343 [M + Na]^+^ | 1065 [M + Na-146-132]^+^  873 [M + Na-470]^+^  769 [(M + Na-132-146-132-18)-146]^+^  595 [M + Na-470-132-146]^+^  493 [470+Na]^+^  1039 [M + HCOO-146-132-18]^-^ | Yemuoside YM21 |
| 13 | 31.1 | C_58_H_92_O_25_ | 1233 [M+HCOO]^-^  1211 [M + Na]^+^ | 1065 [M + Na-146]^+^  933 [(M + Na-146-132]^+^  597 [M + Na-146-132-146-162-28]^+^  475 [470+Na-18]  347 [146+162+16+Na] | Yemuoside YM_10_ |
| 14 | 33.2 | C_57_H_90_O_25_ | 1219 [M+HCOO]^-^  1197 [M + Na]^+^ | 849 [M-H-324]^-^  609 | Yemuoside YM24. |
| 15 | 45.0 | C_45_H_70_O_16_ | 911 [M+HCOO]^-^  889 [M + Na]^+^ | 611 [M + Na-146-132]^+^  567 [M + Na-146-132-44]^+^  587 [M –H-146-132]^-^  455 [M –H-146-132-132]^-^ | Yemuoside YM37 |
| 16 | 48.9 | C_51_H_82_O_20_ | 1059 [M+HCOO]^-^  1037 [M + Na]^+^ | 585 [M –H-146-132-132-18]^-^ | new |
| 17 | 50.4 | C_47_H_74_O_18_ | 925 [M-H]^-^  949 [M + Na]^+^ | 773 [M + Na-176]^+^  749 [M-H-176]^-^  603 [M-H-176-146]^-^  471 [M-H-176-146-132]^-^ | new |
| 18 | 53.7 | C_41_H_66_O_12_ | 795 [M+HCOO]^-^  751 [M + H]^+^ | 749 [M-H]^-^ | 3-O-α-L-rhamnopyranosyl-(1→2)-α-L-arabinopyranosyl-hederagenin |
| 19 | 55.7 | C_45_H_70_O_15_ | 849 [M-H]^-^  873 [M + Na]^+^ | 717 [M-H-132]^-^  571[M-H-132-146]^-^  439[M-H-132-146-132]^-^ | 3-O-α-L-arabinopyranosyl-(1→3)-α-L-rhamnopyranosyl-(1→2)-α-L-arabinopyranosyl-Akebonic acid |
| 20 | 60.0 | C_47_H_74_O_17_ | 909 [M - H]^-^  933 [M + Na]^+^ | 757 [M + Na-176]^+^  611 [M+ Na-176-146]^+^  455 [M-H-176-146-132]^-^ | 3β-[(*O*-β-D-glucuronopyranosyl-(1→3)-*O*-[α-L-rhamnopyranosyl-(1→2)]-α-L-arabinopyranosyl)oxy]olean-12-en-28  -oic acid |
